# Discovery of the First Insect Nidovirus, a Missing Evolutionary Link in the Emergence of the Largest RNA Virus Genomes

**DOI:** 10.1371/journal.ppat.1002215

**Published:** 2011-09-08

**Authors:** Phan Thi Nga, Maria del Carmen Parquet, Chris Lauber, Manmohan Parida, Takeshi Nabeshima, Fuxun Yu, Nguyen Thanh Thuy, Shingo Inoue, Takashi Ito, Kenta Okamoto, Akitoyo Ichinose, Eric J. Snijder, Kouichi Morita, Alexander E. Gorbalenya

**Affiliations:** 1 Department of Virology, National Institute of Hygiene and Epidemiology, Hanoi, Vietnam; 2 Department of Virology, Institute of Tropical Medicine, Global COE Program, Nagasaki University, Nagasaki, Japan; 3 Molecular Virology Laboratory, Department of Medical Microbiology, Leiden University Medical Center, Leiden, The Netherlands; 4 Department of Biochemistry, Graduate School of Medical Science, Nagasaki University, Nagasaki, Japan; 5 Central Laboratory, Institute of Tropical Medicine, Nagasaki University, Nagasaki, Japan; University of North Carolina-Chapel Hill, United States of America

## Abstract

Nidoviruses with large genomes (26.3–31.7 kb; ‘large nidoviruses’), including *Coronaviridae* and *Roniviridae*, are the most complex positive-sense single-stranded RNA (ssRNA+) viruses. Based on genome size, they are far separated from all other ssRNA+ viruses (below 19.6 kb), including the distantly related *Arteriviridae* (12.7–15.7 kb; ‘small nidoviruses’). Exceptionally for ssRNA+ viruses, large nidoviruses encode a 3′-5′exoribonuclease (ExoN) that was implicated in controlling RNA replication fidelity. Its acquisition may have given rise to the ancestor of large nidoviruses, a hypothesis for which we here provide evolutionary support using comparative genomics involving the newly discovered first insect-borne nidovirus. This Nam Dinh virus (NDiV), named after a Vietnamese province, was isolated from mosquitoes and is yet to be linked to any pathology. The genome of this enveloped 60–80 nm virus is 20,192 nt and has a nidovirus-like polycistronic organization including two large, partially overlapping open reading frames (ORF) 1a and 1b followed by several smaller 3′-proximal ORFs. Peptide sequencing assigned three virion proteins to ORFs 2a, 2b, and 3, which are expressed from two 3′-coterminal subgenomic RNAs. The NDiV ORF1a/ORF1b frameshifting signal and various replicative proteins were tentatively mapped to canonical positions in the nidovirus genome. They include six nidovirus-wide conserved replicase domains, as well as the ExoN and 2′-O-methyltransferase that are specific to large nidoviruses. NDiV ORF1b also encodes a putative N7-methyltransferase, identified in a subset of large nidoviruses, but not the uridylate-specific endonuclease that – in deviation from the current paradigm - is present exclusively in the currently known vertebrate nidoviruses. Rooted phylogenetic inference by Bayesian and Maximum Likelihood methods indicates that NDiV clusters with roniviruses and that its branch diverged from large nidoviruses early after they split from small nidoviruses. Together these characteristics identify NDiV as the prototype of a new nidovirus family and a missing link in the transition from small to large nidoviruses.

## Author Summary

Research in virology is driven towards the characterization of a limited number of socioeconomically important pathogens, mostly those infecting humans. Yet, characterization of other viruses may advance our understanding of these topical pathogens and the fundamentals of virology. Here we describe the discovery of a virus of unknown clinical relevance that has many remarkable features. The virus was coined Nam Dinh virus (NDiV) after a Vietnamese province. It is a mosquito-borne virus with a 20.2 kilobase genome, the largest among non-segmented single-stranded RNA viruses of insects. Employing bioinformatics tools, we show that NDiV prototypes a new family and is a missing evolutionary link connecting the distantly related nidoviruses with small and large genomes, including important and diverse pathogens such as porcine respiratory and reproductive syndrome virus (∼15-kilobase genome) and SARS coronavirus (∼30 kilobases), respectively. NDiV and large nidoviruses form a phylogenetic cluster and share a set of core replicative enzymes. They exclusively encode an exoribonuclease that presumably controls replication fidelity. Its acquisition may have promoted the emergence of viruses with single-stranded RNA genomes larger than ∼20 kilobases. This study highlights the benefits of broad virus discovery efforts for fundamental and applied research.

## Introduction

**Figure 1:**
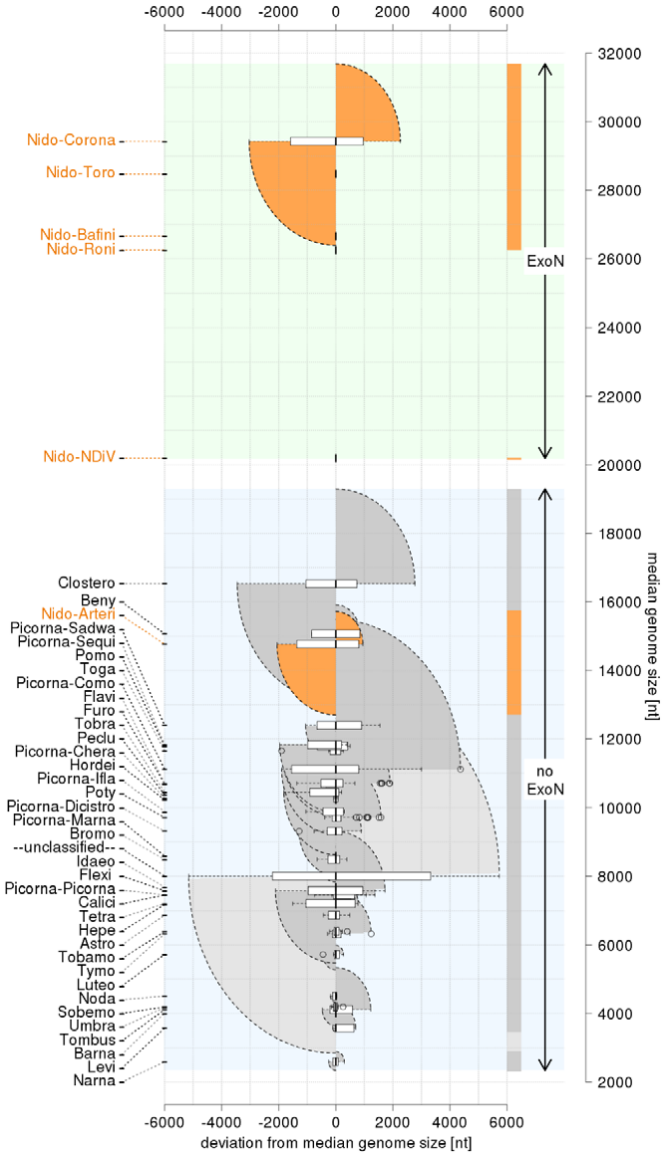
Distribution of positive-sense single-stranded RNA virus genome sizes. The *Coronaviridae* is split into the corona- and toro-/bafinivirus groups. Prefix Nido- and Picorna- are for *Nidovirales* and *Picornavirales*, respectively. Group specific box-whisker plots are aligned along the X-axis by their medians (bold line) normalized to zero. The box spans from the first to third quartile; the whiskers (dashed lines) are <1.5 times the inter-quartile range; outliers are circles. Families/groups are ranked by median genome size along Y-axis. Genome size ranges are colored by semi-circles: nidoviruses (dark orange), other classified (dark grey) and unclassified viruses (light grey). Three non-overlapping zones regarding the presence of the exoribonuclease (ExoN) are highlighted in the genome size distribution (from top to bottom): ExoN-encoding large nidoviruses and NDiV (light green); in-between not-sampled size zone (white); ExoN-lacking ssRNA+ viruses (light blue).

Viruses employing positive-sense, single-stranded RNA genomes (ssRNA+) form the most abundant class and its members are known to infect all types of hosts except *Archaea*. They have evolved genome sizes in the range of ∼3.0 to 31.6 kb (Fig. 1). This size range is the largest among those of the different classes of RNA viruses, although it is small compared to those of DNA viruses and cellular organisms. These profound genome size differences between RNA and DNA life forms are inversely correlated with mutation rates, which are highest in RNA viruses, thought due to the lack of proofreading during replication [1]–[3].

Recently, the molecular basis of the relation between RNA virus genome sizes and mutation rates has been revisited in studies of nidoviruses with large genomes (“large nidoviruses”). These viruses, with genomes of 26.3 to 31.6 kb, include the *Coronaviridae* and *Roniviridae* families and are at the upper end of the RNA virus genome size range [4]. They are uniquely separated from other ssRNA+ viruses (3.0–19.6 kb genomes), including the distantly related *Arteriviridae* family (12.7–15.7 kb genomes; “small nidoviruses”) with which they form the order *Nidovirales* [4]–[6]. The order includes five major lineages of viruses that infect vertebrate and invertebrate hosts. Their complex genetic architecture includes multiple open reading frames (ORFs) that are expressed by region-specific mechanisms. The first two regions are formed by the two 5′-most and partially overlapping ORFs, ORF1a and ORF1b, which are translated from the genomic RNA to produce polyproteins 1a (pp1a) and pp1ab. The expression level of ORF1b is downregulated relative to that of ORF1a by the use of the ORF1a/1b ribosomal frameshifting signal [7], [8]. Both pp1a and pp1ab are autoproteolytically processed by ORF1a-encoded proteases to yield numerous products that control genome expression and replication [9]. The third, 3′-located region of the nidovirus genome includes multiple smaller ORFs (3′ORFs), although the number of these ORFs varies considerably among nidoviruses. These genes are expressed from 3′-coterminal subgenomic mRNAs to produce the structural proteins incorporated into the enveloped nidovirus particles and, optionally, other proteins modulating virus-host interactions [10]–[13]. With the exception of a few nidoviruses, the subgenomic and genomic mRNAs are also 5′-coterminal. A mechanism of discontinuous negative-stranded RNA synthesis, yielding the templates for subgenomic mRNA production, is thought to control this mosaic structure of nidovirus mRNAs. The synthesis of subgenome-length negative stranded RNAs is guided by short transcription-regulating sequences (TRSs) – located in the common “leader sequence” (near the genomic 5′ end) and in each “mRNA body” (upstream of the expressed ORFs) - that share a conserved core sequence and flank the genome region that is *not* present in the respective subgenomic mRNAs.

The nidovirus ORF1b encodes key replicative enzymes whose number and type vary between the major nidovirus lineages. They invariably include an RNA-dependent RNA polymerase (RdRp) and a superfamily 1 helicase (HEL1) [14], which are most common in other RNA viruses, and several other RNA-processing enzymes that are either unique to nidoviruses (uridylate-specific endonuclease (NendoU) and 3′-to-5′exoribonuclease (ExoN)) or rarely found outside nidoviruses (2′-*O*-methyltransferase (OMT); [4]). Among these enzymes, the ExoN domain has properties that are most relevant for understanding the relation between genome size and mutation rate in RNA viruses.

Bioinformatics-based analysis originally identified the ExoN domain only in the genomes of large nidoviruses and mapped it in the vicinity of HEL1, a key replicative enzyme [15]. It also revealed a distant relationship between ExoN and a cellular DNA-proofreading enzyme. Based on these observations, nidoviruses were proposed to have acquired ExoN to control the replication fidelity of their expanding genome [15]. The enzymatic activities of ExoN were subsequently verified and detailed in biochemical studies [16], [17]. Likewise, and in line with the expectations, ExoN-inactivating mutations were shown to decrease RNA replication fidelity by ∼15–20 fold in two coronaviruses, mouse hepatitis virus (MHV) and SARS coronavirus (SARS-CoV), while only modestly affecting virus viability [18], [19]. These results strongly support a critical role of ExoN in the control of replication fidelity of large nidoviruses, although more mechanistic insight is clearly required before the current paradigm connecting RNA virus mutation rates and genome size control could be definitively revised to include proof-reading during the replication of large RNA genomes [20].

Major advancements toward this goal are expected to come from studies of the structure and function of ExoN, which aim to elucidate the molecular mechanism of its action. In addition, genomics studies could contribute to this quest by providing insights into the role of ExoN in RNA virus evolution. Accordingly, if ExoN was acquired to ensure the expansion of RNA genomes beyond a certain size, we may expect (i) a genome size threshold that separates RNA viruses with and without ExoN; (ii) *all* nidoviruses with genome sizes above this threshold to encode ExoN; and (iii) no other domain than ExoN to correlate, functionally and phyletically, with genome size control in large nidoviruses.

In this respect, the characterization of nidoviruses with a genome size in the gap that currently separates small and large nidoviruses should, in theory, be particularly insightful. However, whether these viruses actually exist has thus far remained an open question. Three considerations suggest that if nidoviruses with intermediate-sized genomes ever evolved they may already have gone extinct. First, it is recognized that the evolution of RNA viruses is characterized by a high birth-death rate and the extinction of numerous virus lineages, resulting in the fast turnover of species [21]. Secondly, the genome size gap between large nidoviruses and all other known ssRNA+ viruses has existed without exception since genome sequencing began in the 1980s. As of the late 1980s, this gap has been bordered by closteroviruses (from the bottom) and nidoviruses (from the top) (Fig. 1). Likewise and thirdly, all nidovirus genomes sequenced to date have sizes that are similar to either IBV (27,600 nt) [22] or EAV (12,700 nt) [23], which were the first fully sequenced coronavirus and arterivirus genomes, respectively. The evident under-representation of RNA viruses with relatively large genomes is even more striking in the light of the continuous flow of newly identified ssRNA+ viruses with smaller genome sizes [24] (Fig. 1).

In sharp contrast to these considerations and prior observations, we here report the discovery of a nidovirus with a genome size that is intermediate between those of small and large nidoviruses. This elusive and precious evolutionary link is an insect-borne virus with the largest ssRNA+ genome for any insect virus known to date. Comparative genome analyses involving this newly identified virus provide evolutionary evidence for the acquisition of the ExoN domain by a nidovirus (ancestor) with a genome size in the range of ∼16–20 kb. This range appears to define the size limit for the expansion of ssRNA+ virus genomes, which may be achieved in evolution without the recruitment of a specialized enzyme that controls replication fidelity. Furthermore, we found that two other replicative enzymes, N7-methyltransferase (NMT) and NendoU, are not encoded by toroviruses and invertebrate nidoviruses, respectively, indicating that they may contribute “optional activities” for the nidovirus replication machinery. Together our results highlight the broad benefits of virus discovery efforts applied to mosquitoes.

## Results

### Virus field study

In Vietnam, between 2,000 and 3,000 cases of acute encephalitis syndrome (AES) are reported annually, of which about 40% are confirmed to be associated with Japanese encephalitis virus (JEV). The etiological agent(s) in the other 60% of cases remains unknown [25], but they share demographic characteristics and seasonality with the JEV cases. Hence, the involvement of other arboviruses in non-JE AES was postulated and the virus described in this paper was identified in search of such pathogens, which may infect both humans and mosquitoes.

**Figure 2:**
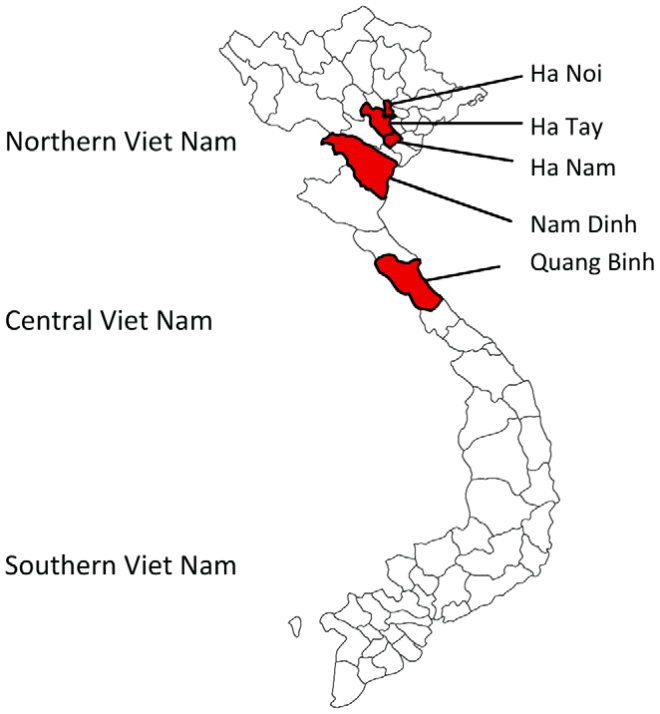
Map of the Vietnam provinces, where mosquito surveillance was conducted between 2001 and 2003.

During continued JEV surveillance between September 2001 and December 2003, 359 pools containing one of six mosquito species (see Materials and Methods) were collected indoors in Northern and Central Vietnam at one- to three-month intervals. The study areas included Hanoi and other cities located in the provinces of Ha Nam (Chuyenngoan, Mocbac), Ha Tay (Catque, Phuman and Chuongmy), Nam Dinh, and Quang Binh (Fig. 2). The majority of Catque inhabitants are farmers who cultivate rice in watered paddy fields and raise pigs. Phuman and Quangbinh, however, are highlands.

### Discovery of the first mosquito-borne nidovirus having an intermediate genome size

Mosquito pools were tested for the presence of viruses using infection of different cell lines as a read-out assay. Homogenates that were prepared from some pools containing *Culex tritaeniorhynchus* and *Culex gelidus* induced cytopathic effects in the C6/36 mosquito cell line. Most of these were attributed to JEV (24 different strains; data not shown), but for 10 specimens a routine laboratory screening for JEV and other circulating flaviviruses (such as Dengue and West Nile viruses) by RT-PCR and/or serology yielded negative results.

**Figure 3:**
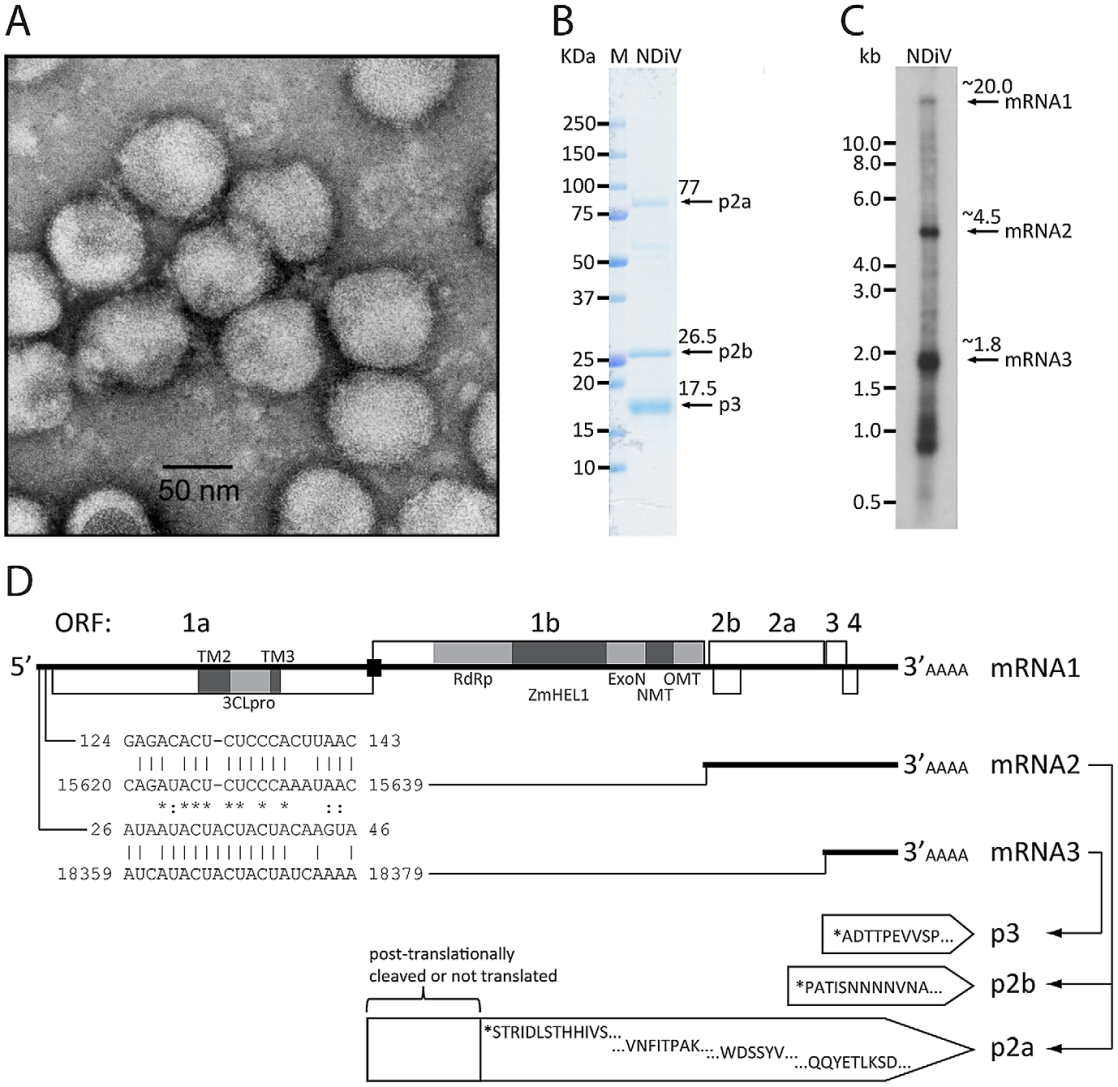
NDiV characteristics. (A) Electron micrograph of negatively stained NDiV virions. (B) SDS-PAGE analysis of NDiV virion proteins. (C) Detection of NDiV genomic (mRNA1) and subgenomic mRNAs (mRNAs 2 and 3) by Northern blot hybridization analysis of total intracellular RNA from virus-infected cells, using a radiolabeled probe complementary to the 3′end of the NDiV genome. (D) NDiV genome organization and expression. Open reading frames (ORFs) are represented by open rectangles and ORF1a- and ORF1b-encoded protein domains identified by bioinformatics analyses (see Table 2) are highlighted in grey. Peptide sequences of virion proteins were determined as described in the Materials and Methods section and mapped to the products of ORFs 2a, 2b, and 3 (bottom-right). N-terminal protein sequences are indicated by (*), other peptide sequences indicate inner sequences. The actual molecular size of the ORF2a product (approximately 77 KDa in SDS-PAGE in B) is considerably shorter than the calculated size (102 KDa), suggesting that p2a may be post-translationally proteolytically processed or that its translation starts at another AUG codn in the ORF. Two pairs of conserved potential TRSs – for sg mRNAs 2 and 3, respectively - were identified in the NDiV genome and aligned (bottom-left), with each pair consisting of a putative leader TRS in the 5′-UTR and a body TRS in the 3′-proximal region of the genome. Between these TRS pairs, eight and three positions include complete match (*) and nucleotide overlap (:), respectively.

Subsequently, infected culture fluid (ICF) from cells infected with unknown agents were analyzed by electron microscopy, which revealed an enveloped virus with a diameter of 60–80 nm (Fig. 3A). This virus was named Nam Dinh virus (NDiV), after the geographic locality of its first apparent isolation, although this origin could not be confirmed later on. However, for historical reasons, this name was retained for all subsequent isolates, and the analysis of one of those (02VN178) is described here. NDiV was identified in four mosquito pools, two from *Culex vishnui* and two from *Culex tritaeniorhynchus*, collected in two other provinces of Vietnam (Table 1). PCR amplification using virus-specific primers to an ORF1b region (see below) was employed to verify the presence of NDiV in the mosquito samples, but to date no other insects have been probed for the presence of the virus. It also remains to be investigated whether NDiV causes disease in susceptible hosts and whether it may infect humans.

**Table 1:** Mosquito pools collected in Vietnam from which NDiV was isolated.

pool^a^	location	month	Species	quantity^b^
02VN009	Ha Tay	Mar	*Culex vishnui*	25
02VN018	Quang Binh	Mar	*Culex vishnui*	170
02VN178	Quang Binh	Aug	*Culex tritaeniorhynchus*	102
02VN180	Quang Binh	Aug	*Culex tritaeniorhynchus*	83

a 359 mosquito pools were collected between Sep. 2001–Dec.2003. The four pools listed in the table were collected in 2002 and infected also with Banna virus.

b number of mosquitoes in each pool.

Purified NDiV was used for virion protein analysis (Fig. 3B) and genome sequencing (Fig. S1; Materials and Methods). *In silico* translation of the unsegmented, 20,192 nt-long NDiV genome (GenBank accession number DQ458789) indicated that it contained at least six ORFs: ORF1a (nt 361–7869), ORF1b (7830–15635), ORF2a (15660–18356), ORF2b (15674–16309), ORF3 (18402–18875) and ORF4 (18754–19101) (Fig. 3D). The region encompassing ORFs 3 and 4 also contains a few smaller potential ORFs. The coding region of the genome is flanked by a 5′-untranslated region (UTR) (1–360) and a 3′-UTR (19102–20192), with the latter being followed by a poly(A) tail. The 5′-UTR includes two AUG codons indicating that translation initiation for ORF1a/ORF1b is likely mediated by another mechanism than ribosomal scanning. Three pairs of ORFs (1a–1b, 2a–2b, and 3–4) overlap to variable degrees; particularly, ORF1b overlaps ORF1a in the −1 frame (Fig. 3D; see also below). Overall, these results showed that NDiV is an insect-borne ssRNA+ virus with the largest genome known so far - twice the size of the next largest one, which is the genome of the *Iflavirus* Brevicoryne brassicae picorna-like virus [26] (Fig. 1).

The NDiV genome organization most closely resembles that of nidoviruses, the only group of ssRNA+ viruses that includes representatives with genomes larger than that of NDiV. This putative relationship was subsequently verified in experimental and bioinformatics analyses of the function and expression of the 3′-ORFs region and in bioinformatics analyses of ORF1a and ORF1b, as described below. The latter studies also provided insights into the evolution and molecular biology of other nidoviruses.

### Function and expression of the 3′-ORFs region

Three virion proteins, p2a, p2b, and p3, were assigned to ORFs 2a, 2b, and 3, respectively, by peptide sequencing analysis (Fig. 3D). No significant similarity was found between these ORFs of NDiV and proteins of other origin in BLAST-mediated searches [27]. The p2b protein is highly hydrophilic and enriched with proline (7.5%) and acidic residues (17.8%), and – relative to other virion proteins – with basic residues (7.9%) making it a potential nucleocapsid (N) protein. The p2a and p3 proteins, and the putative protein encoded in ORF4 (p4) contain, respectively, six, two, and two stretches of hydrophobic residues indicative of transmembrane helices (Fig. S2). These proteins also include, respectively, twelve, two, and three potential N-linked glycosylation signals (NXS/T), and fifteen, six, and four cysteine residues that might form disulfide bridges at locations flanked by hydrophobic regions. These characteristics are typical for glycoproteins of other RNA viruses. Based on size considerations, the largest protein, p2a, might be an equivalent of the spike (S) protein, while p3 and/or p4 might be a smaller glycoprotein and an equivalent of the membrane (M) protein of nidoviruses.

We also asked whether NDiV resembles other nidoviruses in using subgenomic mRNAs for expressing the 3′-end ORFs located downstream of ORF1b. First, we attempted to identify potential TRS motifs in the viral genome sequence, which were expected to reside in the 5′-UTR as well as in the regions immediately upstream of ORF2a, 3, and 4. Although no common repeats larger than six nucleotides were identified in these four areas, we noticed the presence of two pairs of near-perfect repeats: the first pair located in the 5′-UTR (nt 26–40 of the genome) and the region upstream of ORF3 (14 out of 15 residues are identical), and the second pair encompassing nt 125–137 of the 5′-UTR and a sequence immediately upstream of ORF2a/2b (12 out of 13 residues are identical) (Fig. 3D). The two pairs share from ∼43 to 52% pair-wise sequence identity in an alignment containing a single gap (Fig. 3D), and no other repeats of comparable or larger size were found in the analyzed areas. The locations and sizes of these repeats suggest they are TRS signals, although no candidate TRS was identified immediately upstream of ORF4; to our knowledge, the use of two alternative leader TRSs has not been observed in other nidoviruses thus far. These observations suggested that NDiV uses at least two subgenomic mRNAs for the expression of the 3′-located ORFs and that these mRNAs have 5′-terminal sequences of different size in common with the viral genome.

To verify this model, we used a P^32^-labelled probe complementary to the 3′-end of the NDiV genome in a Northern blot hybridization with total RNA isolated from NDiV–infected C6/36 cells (see Materials and Methods; Table S2, and Fig. 3C). This analysis revealed three prominent RNA species with apparent sizes of about 20, 4.5, and 1.8 kb, which match those expected for the genomic RNA and two subgenomic RNAs, mRNA2 (to express ORF2a and ORF2b) and mRNA3 (for ORF3 and possibly ORF4), respectively. We also observed a set of less abundant bands in the 0.9–1.1-kb size range, whose origin(s) and relevance remain to be established.

### ORF1a/ORF1b ribosomal frameshift signal

**Figure 4:**
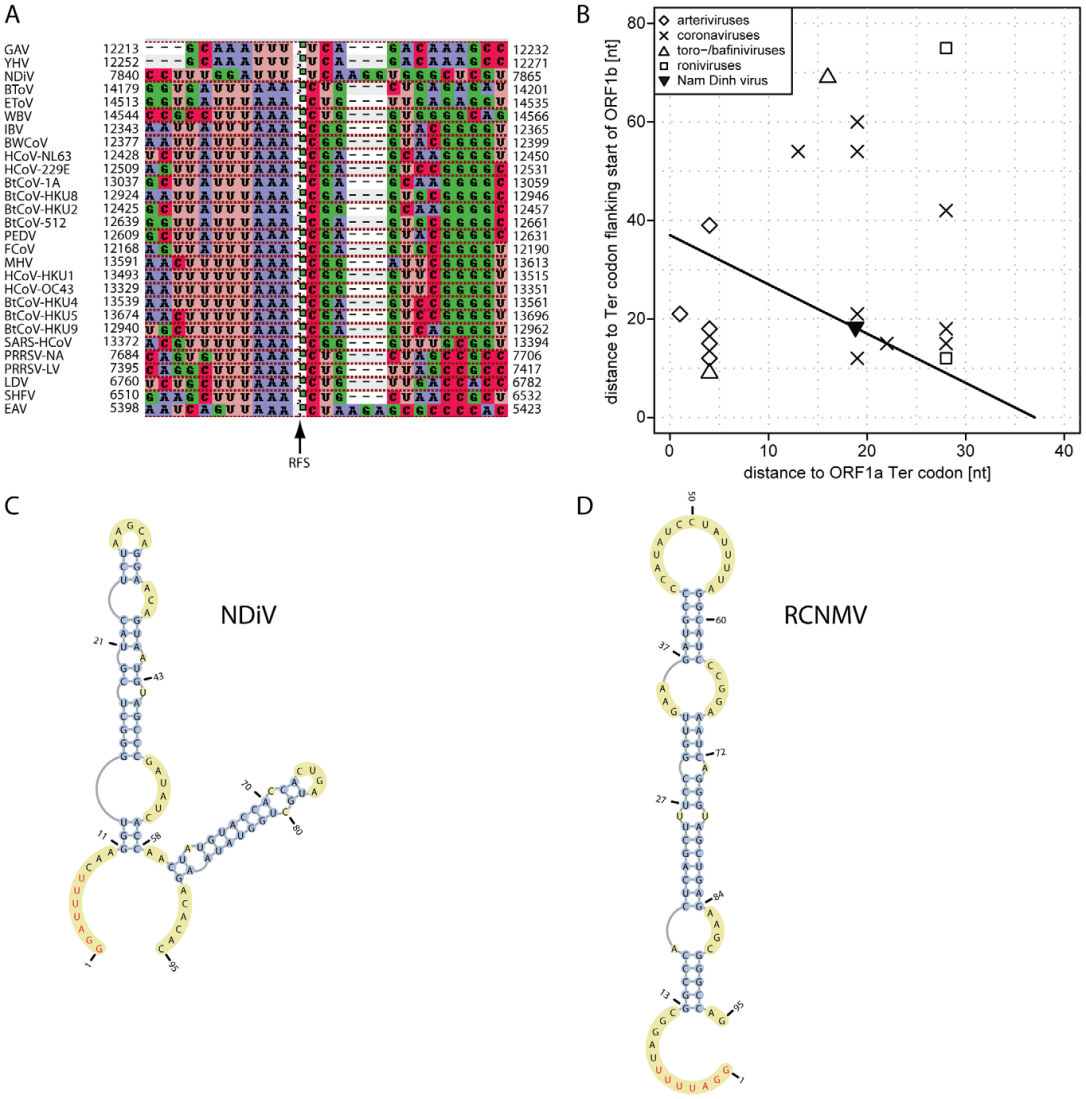
ORF1a/ORF1b ribosomal frameshifting in the NDiV genome. (A) The nucleotide alignment around the open reading frame (ORF) 1a/1b ribosomal −1 frameshift (RFS) in 28 nidovirus species. The alignment column marked with an arrow indicates the RFS position and precedes a nucleotide which is read twice, as the third nucleotide of an ORF1a codon and the first coding nucleotide of ORF1b, respectively. In NDiV this residue is predicted to be located at genome position 7851. Numbers flanking the alignment indicate genomic positions. (B) RFS position in the ORF1a/ORF1b overlap of nidoviruses. The RFS position in selected nidoviruses is plotted as distances between RFS and termination codons flanking ORF1b and ORF1a from upstream and downstream, respectively. In NDiV, the predicted RFS (filled triangle) and all other candidate positions are located on black line in the 40 nt ORF1a/ORF1b overlap. (C and D) RNA secondary structure of sequence fragments around of the ORF1a/ORF1b overlap in NDiV and Red clover necrotic mosaic virus (RCNMV) as predicted by pknotsRG [30]. The putative slippery sequences are in red (see also A). The predicted stem loop in RCNMV closely matches the one presented in [31].

Nidoviral ORF1a/ORF1b −1 ribosomal frameshifting (RFS) is controlled by a “slippery sequence” and a stem-loop or pseudoknot RNA structure immediately downstream [7]. RFS is conserved in nidoviruses and this property is widely used for computational mapping of its determinants in newly sequenced genomes. We followed this approach to map potential RFS signals in the NDiV genome (Fig. 4). The 40-nt NDiV ORF1a/ORF1b overlap region was found to have the best match (GGAUUUU) with the slippery sequence (AAAUUUU) of invertebrate roniviruses [28], which deviates considerably from the pattern (XXXYYYZ) conserved in vertebrate nidoviruses (Fig. 4A). No appreciated similarity with the latter motif was found in the NDiV ORF1a/ORF1b overlap region. The distances separating the NDiV putative RFS from the termination codons flanking the ORF1a/ORF1b overlap are within the range found in large nidoviruses, while being out of the distance range to the ORF1a stop codon of small nidoviruses (Fig. 4B). According to the analysis of a 190-nt sequence - which starts within the NDiV ORF1a/ORF1b overlap - with Mfold [29] and pknotsRG [30], the predicted slippery sequence is followed by a complex stem-loop structure; no pseudoknots, unless forced, are predicted in this region (Fig. 4C). The slippery sequence, distance to the downstream RNA secondary structure, and predicted fold resemble those of Red clover necrotic mosaic virus (RCNMV), a ssRNA+ plant virus of the family *Tombusviridae* [31], [32] (Fig. 4C–D). These results identified the critical elements of the putative NDiV RFS as being most unique among those described for members of the order *Nidovirales*.

### Nidovirus-wide conserved domains: TM, 3CLpro, RdRp, Zm-HEL1, and NendoU

Nidoviruses are distinguished from other RNA viruses by a constellation of 7 conserved domains having the order TM2-3CLpro-TM3-RdRp-Zm-HEL1-NendoU, with the first three being encoded in ORF1a and the remaining four in ORF1b. TM2 and TM3 are transmembrane domains, Zm is a Zn-cluster binding domain fused with HEL1, and 3CLpro is a 3C-like protease [4] (however see below). Since NDiV was found to be very distantly related to the other nidoviruses known to date, sequence-based functional characterization presented a considerable technical challenge. In comparative sequence analysis, profile-based methods that employ multiple sequence alignments are known to achieve the best signal-to-noise ratios [27], [33], [34]. They have been the methods of choice for establishing remote relations in biology, also in our prior studies of nidoviruses [15], [35]–[37]. In this study we used profile vs. sequence and profile vs. profile searches as implemented in HMMer and HHsearch, respectively, for general comparisons. To prepare profiles, we selected representatives of small and large nidoviruses, and also three subsets of large nidoviruses (coronaviruses, toro/bafiniviruses, and roniviruses). Using profile-based searches we identified counterparts (orthologs) of nidovirus-wide conserved enzymatic domains in the NDiV pp1ab. For the identification of TM2 and TM3, predictions of transmembrane helices by TMpred were used.

Six out of the seven nidovirus-wide conserved protein domains, TM2-3CLpro-TM3-RdRp-Zm-HEL1, were mapped in the canonical position and order in the NDiV ORF1a/1b sequence (Table 2). Three of these putative NDiV domains, 3CLpro [9], [38], RdRp [39], and HEL1 [40] are enzymes conserved in all nidoviruses [14]. They have counterparts of all invariant and highly conserved residues implicated in catalysis in other nidoviruses, a finding indicative of the functionality of these proteins in NDiV.

**Table 2:** Mapping replicative protein domains on the NDiV genome.

						basis for domain assignment			
nsp^a^^,^ b homolog	start in genome	end in genome	length [aa]	name	description	target^d^	query diversity^e^	method	support^f^
4	3766	4191	142^c^	TM2	transmembrane domain	pp1a	-	TMpred	>500
5	4549	5352	268	3CLpro	3C-like chymotrypsin-like protease^g^	nsp5^g^	roni	HMMer^g^	4e-05^g^
6	5575	5706	44^c^	TM3	transmembrane domain	pp1a	-	TMpred	>500
12	9378	11048	557	RdRp	RNA-dependent RNA-polymerase	pp1b	nido	HMMer	1e-11
13	12177	13388	404	ZmHel1	Zn module+Superfamily 1 Helicase	pp1b	nido	HMMer	7e-12
14	13413	14210	266	ExoN	exoribonuclease	pp1b	corona+toro+roni	HMMer	8e-05
14	14211	14912	233	NMT	N7-methyltransferase^h^	nsp14^h^	corona	HHsearch^h^	6e-05^h^
16	14913	15635	242	OMT	2′-*O*-methyltransferase	pp1b	corona+toro+roni	HMMer	2e-02

a Open reading frame (ORF) 1a and ORF1b nucleotide sequences in NDiV were *in silico* translated to obtain the encoded polyprotein (pp) sequences 1a and pp1ab. To map domains in these polyproteins, we employed *HMMer*, *HHsearch*. The obtained significant hits were mapped back to the genome.

b The proteolytic cleavage sites in the NDiV pp1a/pp1ab remain to be identified. To provisionally assign the mapped domains to mature proteins, the names of non-structural proteins (nsp) in SARS-CoV that are autoproteolytically released from pp1a/pp1ab are shown. Note that all replicative domains mapped in NDiV are located in the canonical positions.

c Sizes of TM2 and TM3 as determined by *TMpred* may correspond only to small portions of the respective nsp4 and nsp6 proteins.

d Portions of pp1ab that were submitted as targets to profiles searches.

e Shown is the virus diversity range of a query domain profile: the subfamilies *Coronavirinae* (corona) and *Torovirinae* (toro), the family *Roniviridae* (roni) and the order *Nidovirales* (nido). The used profiles for 3CLpro, RdRp, ZmHel1, ExoN, NMT and OMT are part of an in-house nidovirus domain profile database. No query (“-”) was used for analyses mediated by *TMpred*.

f E-value for *HMMer/HHsearch* based on a database size of 12000 according to the size of the Pfam (version 24.0, October 2009); *TMpred* score otherwise.

g The assignment was done according to a profile-vs-profile search in local mode of the domain flanked by TM2 and TM3 in NDiV against the respective ronivirus profile.

h The assignment was done according to a profile-vs-profile search in global mode of the domain flanked by ExoN and OMT in NDiV and roniviruses against the respective coronavirus.

Like its orthologs in corona- and roniviruses, the NDiV 3CLpro is predicted to employ a catalytic His-Cys dyad. Its substrate-binding site is predicted to include a conserved His residue which was implicated in controlling the P1 specificity for Glu/Gln residues in other viruses, a hallmark of 3C/3CLpros [41]. Surprisingly, despite this finding, no candidate cleavage sites with the characteristic 3CLpro-specific signatures could be identified in the NDiV pp1a/1ab. Consequently, the sizes of all NDiV replicative domains described in this paper (Table 2) are based on the hit sizes in profile searches and are subject to future refinement. Collectively, these results strongly indicate that NDiV encodes all nidovirus-wide conserved replicase domains except for NendoU (Figure 3D; see also below), thus supporting the classification of NDiV as a nidovirus.

### Conserved domains common to large nidoviruses: ExoN and OMT

**Figure 5:**
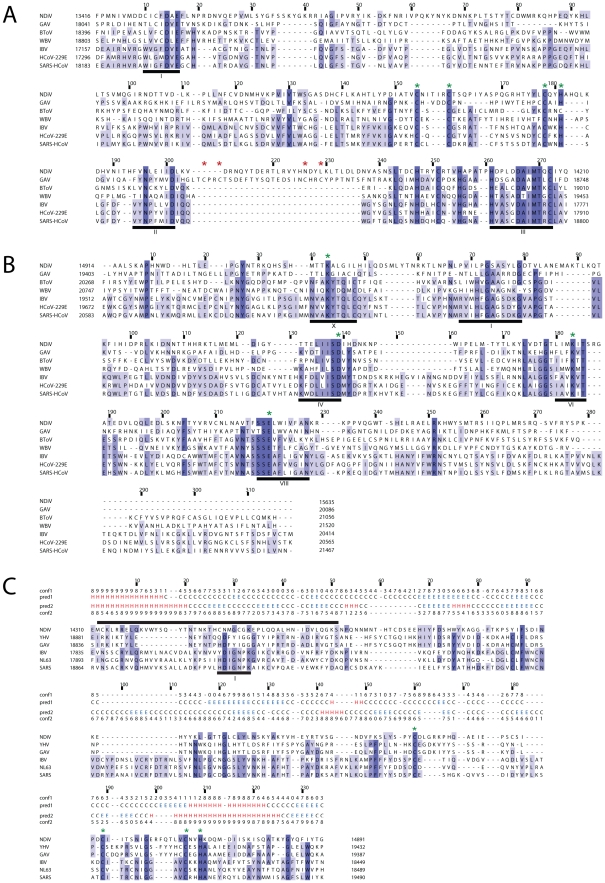
Alignments of ExoN, OMT and NMT domains of NDiV and other nidoviruses. Alignments were compiled utilizing the Muscle program followed by manual inspection. Pictures by JalView [90] and residues are colored according to degree of conservation. Numbers above a column indicate its absolute position in the alignment (start = 1); numbers to the left and to the right of the alignment represent positions in the genome. Selected conserved sequence motifs are highlighted with black bars and roman numbers. (A) In the exonuclease (ExoN) alignment, three motifs are part of the catalytic centre; the domain includes two putative zinc fingers, specific either for roniviruses or for all nidoviruses and highlighted by, respectively, red and green asterisks. (B) In the 2′-*O*-methyltransferase (OMT) alignment, motifs X, IV, VI and VIII include residues of the catalytic tetrad (KDKE, marked with green asterisks) and motif I is involved in binding of the methyl donor [42]. (C) Protein secondary structure predictions by Psipred [86] for the profiles of N7-methyltransferase (NMT) from 3 NDiV/roniviruses (pred1) and 17 coronaviruses (pred2) and corresponding confidence values (conf1, conf2) were added above the alignment. Only 3 coronaviruses, representing alpha- (HCoV NL63), beta- (SARS-CoV) and gammacoronaviruses (IBV), are shown that results in several empty alignment columns. The black bar on top is a region including the methyl-donor binding site (motif I, delineated by [49]) that gave a hit with a functionally similar site of a cellular guanine N7-methyltransferase (fungus *Encephalitozoon cuniculi*) upon HH search of the SCOP database [82] (data not shown). Green asterisks, conserved Cys/His residues that may form a zinc finger.

All large nidoviruses express an ExoN [16] of the DEDD superfamily, which is not found in other ssRNA+ viruses, and an OMT [42], [43] of the RrmJ family, that is not present in arteriviruses [15]. The presence of these domains therefore discriminates large from small nidoviruses. Using profile searches in the ORF1b-encoded part of pp1ab, homologs of these two enzymes were identified in the NDiV genome (Table 2). Using an ExoN multiple sequence alignment of NDiV and large nidoviruses, the conserved motifs I, II, and III, including the catalytic residues (two Asp and one Glu), as well as the ExoN-specific Zn-finger module were identified in the NDiV ortholog (Fig. 5A). Furthermore, the NDiV ExoN shows an insertion whose size and position correspond to those of the second Zn-finger-like module that is exclusively found in roniviruses. However, unlike the ronivirus domain, NDiV appears to lack His/Cys residues potentially involved in Zn-binding. According to a multiple sequence alignment of nidovirus OMTs (Fig. 5B), the putative NDiV OMT contains motifs X, IV, VI and VIII, encompassing residues of the catalytic KDKE tetrad, as well as motif I involved in binding of the methyl donor [42]. These data imply that NDiV ORF1b encodes functional ExoN and OMT domains (Fig. 3D), which are both typical of large nidoviruses.

### Nidovirus- and large nidovirus-specific domains absent in some lineages: NendoU and NMT

NDiV ORF1b includes a ∼750-nt region that is flanked by the upstream ExoN and downstream OMT domains and was expected to encode a NendoU domain [44]–[47], given its presence at this locus in all nidoviruses known so far [15], [48]. Surprisingly, however, profile searches of nidovirus NendoUs revealed no significant hits in the corresponding region of the NDiV sequence (E-values>9.5). This observation prompted us to re-examine the NendoU assignment in other nidoviruses, including the invertebrate roniviruses [15]. Using profile-sequence and profile-profile comparisons mediated by HMMer and HHsearch, respectively, NendoU counterparts were readily identified in all corona-, toro/bafini-, and arteriviruses (E-values<10^−4^), but not in roniviruses (E-values>4.5). We therefore conclude that, unlike other (vertebrate) nidoviruses, the invertebrate NDiV and roniviruses do *not* encode a NendoU domain (Fig. 3D).

We proceeded to analyze this genomic region flanked by ExoN and OMT in invertebrate nidoviruses in more detail. First, using a ronivirus profile vs. NDiV pp1ab sequence comparison, we found that these domains are moderately similar to each other (E-value = 0.18), suggesting a weak conservation of a common function in these newly recognized orthologous domains of NDiV and roniviruses. Their alignment was converted into a profile with which we screened all domains of our in-house nidovirus profile database (see Materials and Methods). Remarkably, the only significant hit (E-value<10^−4^) was recorded against the coronavirus NMT profile (Table 2). For comparison, its similarities with NendoU profiles of corona-, toro/bafini- or arteriviruses were not significant (E-value>1.5). These data indicate that NDiV and roniviruses may encode an NMT domain that is flanked by ExoN and OMT.

The coronavirus NMT domain was originally mapped to the C-terminal half of nsp14 [43], [49]. The corresponding domain in toro/bafiniviruses has a much smaller size (80 aa vs. 200 aa). According to our analysis, it has no significant similarity with the NMT of coronaviruses, or the newly recognized putative NMT of roniviruses and NDiV. Based on these observations, we generated an alignment of the NMT domains of corona- and roniviruses and NDiV (Fig. 5C) in order to search for remote cellular homologs. The N-terminal part of the nidovirus NMT includes a conserved methyl donor binding site (motif I), according to the prior assignment for coronavirus NMTs. In line with this observation, a weak hit between nidovirus NMTs and a cellular guanine N7-methyltransferase involving the motif I region was detected in this study. In their C-terminal part, nidovirus NMTs uniquely include four conserved Cys/His residues indicative of a Zn-binding site that may be part of a separate domain (Fig. 5C).

Collectively these results established a mosaic domain relationship in the pp1ab area flanked by ExoN and OMT domains for large nidoviruses and NDiV. In this genomic region coronaviruses encode both NMT and NendoU domains, while other viruses encode either NendoU (toro/bafiniviruses) or NMT (roniviruses and NDiV).

### Phylogenetic analysis of NDiV and other nidoviruses: challenges and approach

Next, we proceeded to determine the phylogenetic position of NDiV among nidoviruses. The phylogeny was inferred using Bayesian posterior probability trees for a concatenated alignment of three enzymes, 3CLpro, RdRp, and HEL1, that are conserved in all nidoviruses (see Materials and Methods). In line with the current nidovirus taxonomy and genomic data [28], [48], [50], [51], this analysis consistently identified the four known major lineages (arteri-, roni-, corona-, and toro/bafiniviruses), as well as a new one represented by NDiV, as the most deeply rooted branches. Our initial attempts to resolve the relationship among the five lineages produced uncertain results. To address this challenge, we adopted a step-wise approach starting from the analysis of close intra-group relationships in the most abundantly sampled subfamily, *Coronavirinae*, and the family *Arteriviridae*, and finishing with an analysis of the most distant inter-(sub)family relationships between the five major lineages. Prior to the nidovirus-wide phylogenetic analysis, the affinity of arteri-, roni-, and toro/bafiniviruses to the subfamily *Coronavirinae* was evaluated through a profile-based analysis involving conserved domains (see Supplementary Text S1 and Table S1). The obtained results confirmed that the strongest sequence affinity exists between corona- and toro/bafiniviruses, which was evident for the 6 out 8 domains that are conserved between coronaviruses and one or more of the other lineages. The HEL1 was the only domain for which an alternative strongest affinity – between corona- and roniviruses – was documented.

### Unrooted nidovirus phylogeny

**Figure 6:**
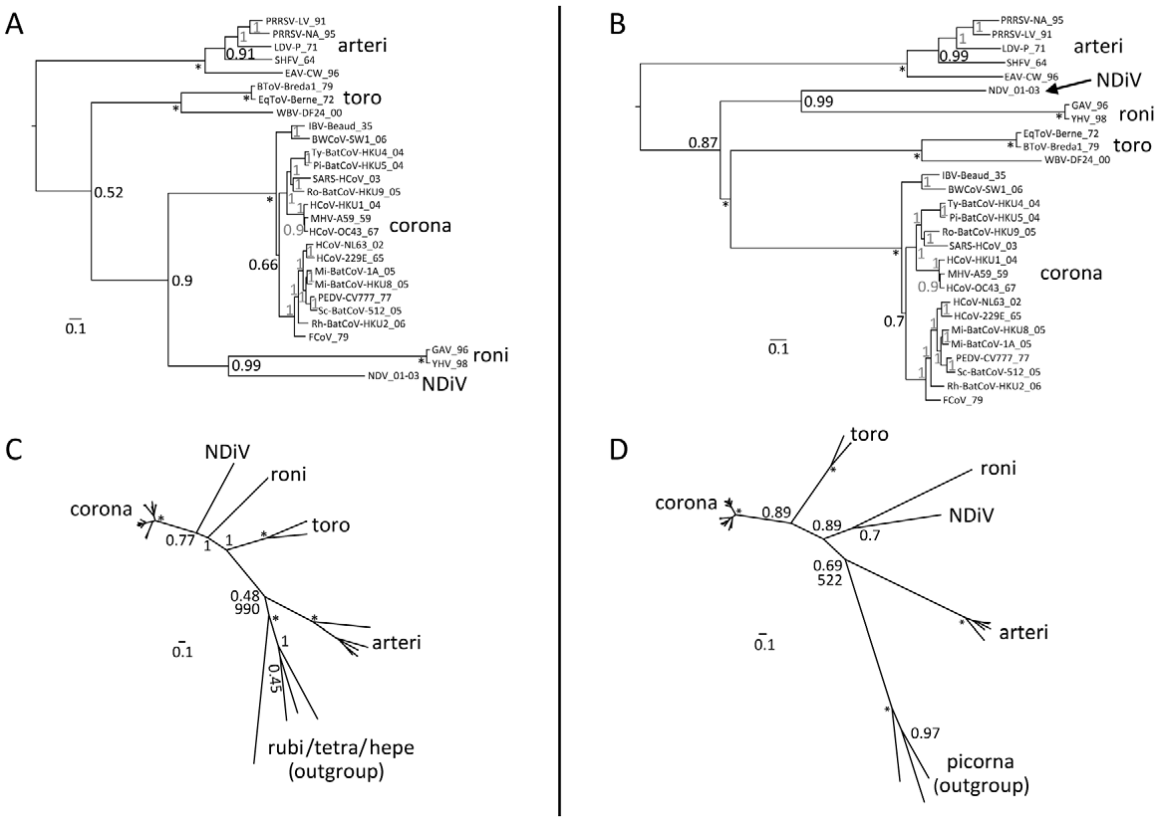
Phylogeny of nidoviruses. To infer phylogenetic relationships between NDiV and other nidoviruses partially constrained trees were calculated using either a concatenated alignment of the three nidovirus-wide conserved domains (A and B) or one nidovirus-wide conserved domain (C and D). For all trees, internal nodes without support value that were fixed prior to the analysis are marked with *, otherwise, numbers indicate posterior probability support values (at the scale from 0 to 1) obtained in either (sub)family- or order-wide analyses (grey and black, respectively). The tree scale bars represent number of substitutions per amino acid position on average. (A) and (B), trees with the constrained topology in which coronaviruses and toro-/bafiniviruses were either fixed as sister clades or not, respectively. Shown are trees for the original alignment which were similar to those obtained for the alignment derivative in which least conserved columns were removed (see Materials and Methods). The trees were rooted on the arterivirus branch. (C) and (D), trees based on conserved HEL1 and RdRp domains, respectively, and including a domain-specific outgroup as described in Materials and Methods. The sister position of coronaviruses and toro-/bafiniviruses was not fixed. For virus listing see trees in A and B. Support values for the outgroup branching in a Maximum-Likelihood analysis with 1000 non-parametric bootstraps, which resulted in an identical topology, is shown below posterior probability support values in both trees. Support values for internal branching within the *Coronavirinae* subfamily and the *Arteriviridae* family are omitted for clarity. The outgroup placement on the arterivirus branch in these analyses was used to root trees in A and B.

The affinity established above was incorporated as prior knowledge in the nidovirus-wide phylogenetic analysis in order to improve the resolution of the most distant relationships. Accordingly, two alternative reconstructions were conducted with the clustering of toro/bafiniviruses and coronaviruses being either fixed or not. When the clustering was not fixed, roniviruses were found to be closest to coronaviruses (Fig. 6A). This topology indicated that the HEL1 sequence affinity dominated over that of the RdRp (Table S1) in the concatenated 3CLpro-RdRp-HEL1 alignment. An alternative nidovirus phylogeny was inferred when the clustering of coronaviruses and toro/bafiniviruses was fixed prior to the inference (Fig. 6B). Importantly, in both trees, NDiV was consistently albeit relatively distantly clustered with roniviruses, indicating that this grouping does not depend on the choice of tree-building parameters and is likely genuine.

### Rooting of nidovirus phylogeny

To infer the direction of nidovirus evolution, we sought to root the nidovirus phylogeny using an outgroup approach. Neither other viruses nor cellular organisms encode the domain constellation that is conserved in nidoviruses, precluding an expansion of the original nidovirus dataset with outgroup sequences to root the tree. This prompted us to split the domain constellation and perform separate analyses of the evolution of the two most conserved nidovirus protein domains, RdRp and HEL1, which are also among the most conserved in ssRNA+ viruses (Fig. 6C–D). Prior to the analysis, major clades comprising coronaviruses, toro-/bafiniviruses, roniviruses, and arteriviruses, and an outgroup were each fixed to be monophyletic.

For the HEL1 tree (Fig. 6C), the part of the alignment covering the most conserved region from motif I to motif VI (see [52]) was used. Representatives of rubiviruses, betatetraviruses, omegatetraviruses, and hepeviruses were used as an outgroup. The resulting topology closely resembles that of the relaxed nidovirus phylogeny (Fig. 6A), in which vertebrate coronaviruses and invertebrate nidoviruses are sister clades, thus confirming that it is dominated by the HEL1-related component.

For the RdRp tree (Fig. 6D), an alignment of the most conserved RdRp region delimited by motifs G and E (see [53]) was used. Representatives of three divergent picornaviruses (an enterovirus, a parechovirus, and a hepatovirus) were used as an outgroup. The resulting topology matches that of the constrained nidovirus phylogeny (Fig. 6B), in which the grouping of corona- and toro-/bafiniviruses was forced, and could thus be considered RdRp-like.

Despite somewhat incongruent topologies in the two protein-specific phylogenies, in both cases the outgroups are consistently placed at the branch leading to arteriviruses, thus separating small- from large- and intermediate-size viruses in nidovirus evolution. The support for the positioning of the outgroups in the RdRp and HEL1 trees by Bayesian/ML estimates (0.69/522 and 0.48/990, respectively) is relatively low and/or varied in analyses by two methods, possibly due to the very large evolutionary distances separating the major virus groups, including the outgroups. We used the rooting on the arterivirus branch to root the nidovirus tree that was inferred using a concatenated alignment of three domains (Fig. 6A–B).

According to this analysis, small nidoviruses are separated from other nidoviruses, and NDiV is monophyletic with roniviruses in a separate clade of invertebrate nidoviruses, which clusters with the group formed by corona- and toro/bafiniviruses. NDiV and roniviruses are separated by a large evolutionary distance indicating that NDiV likely is the prototype of a separate family. The topology of the tree in Fig. 6B is compatible with a scenario in which genome size change during nidovirus evolution was dominated by expansion, with contemporary nidoviruses representing different stages in the transition from small to large ssRNA+ genomes.

## Discussion

We describe the discovery of an insect-borne ssRNA+ virus, called NDiV, possessing a genome organization, virion properties, mRNAs, and putative proteome characteristics that place it in the order *Nidovirales*. In phylogenetic and protein domain analyses NDiV consistently, albeit relatively distantly, clustered with viruses of the family *Roniviridae*, which seems to make sense biologically given that both infect invertebrate hosts. Although the NDiV classification as the first insect nidovirus is beyond doubt, its characterization was only just initiated in this study. NDiV is likely to possess unique properties concerning, for example, the leader-body junctions of its sg mRNAs and the cleavage sites recognized by its 3CLpro, which both require further characterization.

The principal biological significance of the discovery of NDiV is in the intermediate position this virus occupies between small and large nidoviruses in the genome size distribution observed for ssRNA+ viruses. Prior to this study, the existence of currently circulating nidoviruses with genome sizes within this gap was even highly uncertain (see Introduction). Together small and large nidoviruses cover the upper ∼19 kb (∼66%) of the entire ssRNA+ genome size range and are separated by ∼10 kb (32%). The very existence of NDiV validates the previously established evolutionary relationship between the remotely related arteriviruses and coronaviruses that have very different genome sizes [23]. Characterization of arteri- and coronaviruses by comparative genomics has been instrumental in defining the common and unique features of members of the order *Nidovirales* [14], and has guided the delineation of potential targets for antiviral drug design [54].

The inclusion of NiDV in this analysis yields additional and novel insights with implications for nidoviruses and other RNA viruses at large. It allowed us to revise and expand the assignment for two replicative enzymes of nidoviruses – NendoU and NMT. Prior to this study, the former was considered to be a genetic marker of nidoviruses [15]. Still, its (universal) function in the replication cycle of (vertebrate) nidoviruses has remained enigmatic, despite steady progress in the biochemical, structural, and genetic characterization of this enzyme in arteri- and coronaviruses [44]–[47], [55]–[60]. Our analysis showed that invertebrate roniviruses and NDiV do not encode a NendoU domain implying that, contrary to the current paradigm, the utilization of this enzyme in replication may be restricted by the host organism. Surprisingly, and in contrast to the case of NendoU, invertebrate nidoviruses were found to encode a putative NMT, whose ortholog was previously identified in SARS-CoV and shown to be conserved in the subfamily *Coronavirinae* [43], [49]. Our observation indicates that certain aspect(s) of the nidovirus replicative cycle that are controlled by the NMT domain could be similar in coronaviruses and invertebrate nidoviruses, but not toro/bafiniviruses which are otherwise closer to coronaviruses. Collectively, our insights into the phyletic distribution of NendoU and NMT reveal a modularity of some of the major subunits of the replication apparatus in large nidoviruses, which must be rationalized in future mechanistic studies and taken into account in drug development efforts.

Although the NDiV genome size is intermediate between those of small and large nidoviruses, NDiV most closely resembles large nidoviruses in properties that are *not* universally conserved in the order. Particularly, NDiV does not encode a homolog of the replicative protein of unknown function (nsp12) that is exclusively conserved in arteriviruses [14] and it has a set of three replicative enzymes, OMT, NMT, and ExoN, encoded in large but not in small nidoviruses. These three enzymes are encoded in ORF1b, downstream of the RFS (Fig. 3D and Fig. 4) and in the vicinity of the two key enzymes for RNA synthesis, RdRp and HEL1, with their expression level being downregulated relative to that of the ORF1a-encoded subunits.

Despite these common properties, the two methyltranferases (OMT and NMT) differ from ExoN in their relation to genome size. Particularly, OMTs are known to be also encoded by flaviviruses [61] whose genome size of ∼10 kb is average for RNA viruses, while the NMT domain was found to be *lacking* in a subset of large nidoviruses represented by toro-/bafiniviruses (this study). Furthermore, an N-methyltransferase function, albeit associated with a domain seemingly unrelated to the NMT domain of nidoviruses, was identified in the large Alphavirus-like supergroup of ssRNA+ viruses, whose members have genome sizes from ∼7,000 to 19.500 nt [62]–[64]. ssRNA+ viruses use methyltransferases to modify the 5′-end of their mRNAs (cap structure), which was recently found to be essential in the control of translation and innate immunity [65], [66]. It is not clear whether the use of methyltransferases may provide particular benefits for genome size control and/or promote genome expansion, although the involvement of OMT in other modifications than 5′-end capping was previously proposed for large nidoviruses [15].

In contrast to the case of the methyltransferases, the link between ExoN and genome size control in nidoviruses is supported by accumulating evidence obtained from different hypothesis-driven genetic studies [4], [20]. First, ExoN is exclusively found in a phylogenetically compact cluster of ssRNA+ viruses with large genome sizes. Second, cellular homologs of ExoN control the fidelity of replication in DNA-based life forms and are essential to maintain these large genomes. Third, ExoN active site mutants in MHV and SARS-CoV showed a stable phenotype characterized by a clearly enhanced mutation rate and nearly wild-type progeny yields.

The identification of the ExoN-encoding NDiV further strengthens the case for the direct involvement of ExoN acquisition in genome size expansion. First, because of its distant relation to any known virus and its insect host range that is a novelty for nidoviruses, NDiV provides an essentially independent verification for the association of ExoN with RNA viruses employing large genomes.

Second, it increases our confidence that no other domain is associated with large genome sizes in nidoviruses as strongly as ExoN is. The existence of such a domain is unlikely but it cannot be formally excluded because the entire proteomes of nidoviruses are yet to be fully described. However, our confidence about the lack of this alternative domain grows with the decrease of difference between genome sizes of nidoviruses containing and lacking ExoN: the smaller this difference the less capacity remains to encode an additional domain. With the identification of NDiV, this genome size gap decreased from ∼10.6 kb to ∼4.5 kb, the largest drop since this gap could have been recognized (∼14.9 kb in 1991) (Fig. S3).

Third, following the discovery of NDiV, only ∼0.8 kb remains of the other genome size gap of ∼7 kb that previously separated the ExoN-containing nidoviruses from all other ssRNA+ viruses (Fig. 1). Thus, a major step has been made towards a more precise definition of the RNA genome size limit above which the recruitment of a specialized enzyme for replication fidelity control may be a prerequisite. According to a custom binomial test (see Materials and Methods), the probability to observe the association of ExoN and large ssRNA+ genome size by chance may be 10^−6^ or lower. The genome size threshold of ∼20 kb, as defined by NDiV and a closterovirus [67], which has the largest genome size among ssRNA+ viruses other than nidoviruses, is also valid for unsegmented RNA viruses of other classes, all of which do not employ an ExoN in their replicative machinery [21].

**Figure 7:**
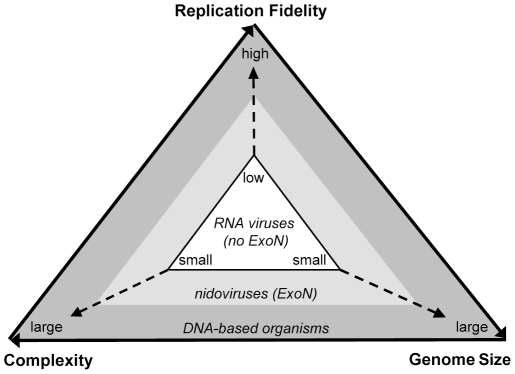
The Eigen trap and a model of nidoviral escape by ExoN acquisition. The scheme depicts the unidirectional relationship between replication fidelity, genome size, and complexity. The vector of variation for the dimensions defined by the three elements of the relationship is shown in a simplified form. The position of RNA viruses in the white triangular space (Eigen trap) and the proposed effect of ExoN acquisition in nidoviruses on this position are indicated.

The fixation of the ExoN domain in nidovirus genomes may be rationalized in the framework of a unidirectional triangular relationship that includes complexity, replication fidelity (mutation rate), and genome size [68] (Fig. 7). In RNA viruses, the low fidelity of replication severely restricts the size of their genomes, which can encode only relatively simple replication complexes that, hence, suffice to support low-fidelity replication [21], [69]. This low-state trap is known as the “Eigen paradox”. Accordingly, a transition from the “low” to the “high” state may not be accomplished by changing only one element of the triangle, e.g. improving replication fidelity, since such a change would not be compatible with the “low” state of the other two elements [68] [70]. The exclusive presence of ExoN in ssRNA+ viruses above 20 kb supports the logic of the Eigen paradox [68]. It also shows how the paradox could be solved with a single evolutionary advancement, the acquisition of ExoN, which may have relieved the constraints on *all* three elements of the triangular relationship (Fig. 7), providing a lasting benefit to the virus lineage that acquired ExoN. This advancement may have been accompanied by an immediate fitness gain. Accordingly, the ExoN acquisition could have provided the ancestral virus with improved control over the fidelity of its replication and the mutation spectrum (quasispecies structure) of its progeny [71], [72], which may have facilitated virus adaptation to the environment [20], [73]. Alternatively, ExoN could have been acquired in an evolutionarily neutral event. Through subsequent mutation this enzyme might have gained beneficial properties for the ancestral virus and its progeny. The functional and structural characterization of known nidoviruses and yet-to-be identified viruses in the genome size range around that of NDiV will be required to clarify this key aspect in the transition from small to large nidoviruses.

The acquisition of ExoN by an ancestral nidovirus must have produced viable progeny but it remains unknown whether, besides ExoN, any additional properties of the ancestral nidovirus were critical for genome expansion, as was speculated elsewhere [15]. Recently an exoribonuclease was identified in the ssRNA- arenaviruses, which have genome sizes below 10 kb [74], [75]. Unlike nidoviruses, arenaviruses employ the exoribonuclease as a domain of their nucleocapsid protein that, accordingly, mediates a non-replicative function. In line with these differences, the nidovirus ExoN and the arenavirus exoribonuclease do not share specific sequence affinity (CL and AEG, unpublished data), indicating that both are likely to have been acquired from independent sources and were integrated into different genetic settings to perform different functions.

NDiV may be the first but likely not the last nidovirus identified in mosquitoes [76]. Systematic probing of these and other insects could lead to the discovery of new nidoviruses, and characterization of those with genomes in the size range between small and large nidoviruses could be particularly insightful. As presented in this study, benefits of these advancements could be multifold and provide a foundation for both fundamental and applied research on newly discovered and already known viruses.

## Materials and Methods

### Mosquitoes handling for virus isolation

During continued surveillance for JEV in Vietnam between September 2001 and December 2003, 24,097 female mosquitoes belonging to six different C*ulex* species (*Culex tritaeniorhynchus*, *Culex gelidus*, *Culex vishnui*, *Culex fusco*, *Culex pseudo*, and *Culex quinquefaciatus*) were collected. They were divided into 359 pools, each containing a single mosquito species and handled with utmost care following the appropriate biosafety measures. For the digestion of blood meals, the samples were kept in 5% glucose for two weeks at room temperature and a humidity of ∼90%. The most abundant species was *Culex tritaeniorhynchus* (10,194 mosquitoes accounting for a 42.3% share), followed by *Culex gelidus* (6,199, 25.7%), *Culex vishnui* (3,780, 15.7%), *Culex quinquefaciatus* (2868, 11.9%), with the remaining species ranging from 0.3%–4.1%. Mosquito pools were stored at −70 C prior to processing for virus isolation.

### Virus propagation in cell cultures

Four cell lines were used to isolate viruses, but NDiV was evident only in samples from *Aedes albopictus* C6/36 cells grown at 28 C in Eagle's Minimum Essential Medium (EMEM) containing 10% fetal calf serum (FCS) and 0.2 mM non-essential amino acids [77]. Pooled mosquitoes were washed three times in sterile phosphate-buffered saline (PBS, pH 7.2) containing 1000 g/ml each of penicillin and streptomycin, followed by rinsing with antibiotics-free PBS. The homogenates were prepared by triturating the mosquitoes in 2%-FCS-EMEM with subsequent centrifugation at 2,000 g for 10 min. The suspensions were filtered (0.22 nm Millipore, USA) and applied to C6/36 cells, which were monitored daily for cytopathic effects, also after three blind passages. The cell death, probably due to apoptosis, was indeed observed upon NDiV infection. The ICF were clarified by centrifugation at 2,000 g for 10 min.

### Genome cloning and sequencing

The nucleic acid was extracted from the purified NDiV virus particles using phenol-chloroform extraction. It migrated as a single band in agarose gel electrophoresis, which was sensitive to RNase but not DNAse treatment, indicative of an RNA virus genome. Accordingly, reverse transcriptase (RT) was used to amplify parts of the NDiV genome by Random Arbitrary Primers-PCR (RAP-PCR) in order to initiate sequence analysis. Cassette primers (C1 and C2) coupled to random hexamers (Hx) were employed. Following synthesis of first and second cDNA strands with C1Hx and C2Hx primers, respectively, PCR amplification was performed using the cassette primers C1 and C2 as per the standard protocol [78]. Three amplicons of different sizes, which were specific for the virus-containing samples, were then cloned in the pCR2.1-TOPO vector (TOPO TA Cloning Kit, Invitrogen) according to the manufacturer's instructions. The sequence of the first cloned fragment (referred to as “index clone”) was determined by Big Dye Terminator Cycle Sequencing using M13 forward and reverse primers in an ABI 310 or 3100 automated DNA sequencer (Applied Biosystems). The cloned region of the genome was extended by ‘gene walking’ using primers based on previously obtained sequence information (Table S2).

To sequence the genomic region upstream of the index clone, the following amplification strategy was used, involving two DNA fragments called double-stranded (ds) cDNA and anchor DNA. To produce ds cDNA, viral genomic RNA was mixed with 10 mM dNTP mix and 2 pmol of 15-mer gene-specific primers (NDiV-RACE492-477RP, NDiV-RACE302-288RPB and NDiV-RACE435-420RPC) (Fig. S1A, Table S2).

An anchor DNA was synthesized by PCR that amplified a specific fragment of pUC19, including its multiple cloning site (Fig. S1B). Both, the ds viral cDNA and PCR product obtained from pUC19 (anchor) were digested by several restriction enzymes whose sites are present in the pUC19 multiple cloning site (*Bam*HI, *Eco*RI, *Kpn*I, *Hind*III, *Sca*I, and *Pst*I). The digested pUC19 PCR products were then purified using the QIAXII gel purification kit (Qiagen) in order to collect the longer DNA fragments. The digested viral cDNAs were also purified by filtration using Micropure-EZ (Millipore) and Microcon YM-100 (Millipore) to remove enzymes and buffers. In a next step, the purified cDNAs and anchor DNAs were mixed and ligated using T4 DNA Ligase (TaKaRa). The unknown region of viral cDNA was then amplified by semi-nested PCR using LA-taq (TaKaRa), two viral gene specific primers and one pUC19 primer (Table S2) as shown in Fig. S1C. The reaction process included an initial denaturation at 96°C for 5 min, 35 cycles at 96°C for 30 sec, 53°C for 30 sec, and 72°C for 7 min, and a final extension at 72°C for 10 min.

The known viral genome sequence was further extended by long RT-PCR which resulted in an 8 kb fragment with a 68-nucleotide polyA tail representing the 3′-end of the NDiV genome. The GeneRacer™ Kit (Invitrogen) was used to sequence the 5′-end of the NDiV's genome.

The NDiV origin of newly obtained sequences was further validated by probing different samples with a primer pair designed against the index clone. This pair of primers recognized NDiV isolates, but not JE and dengue viruses (flaviviruses) or SARS-coronavirus (Coronavirus). These results indicated that NDiV is a novel mosquito virus.

### RNA probe generation for Northern blotting analysis

Specific primers encompassing NDiV nts 19,733 and 20,126 (including 2 Adenines of the poly (A) tail), respectively, were designed (Table S2). The generated PCR product was purified using the Qiaex II gel extraction kit (500) (Qiagen) following the manufacturer's instructions. The purified PCR product was then ligated to a 3.5 kb plasmid (PCR-XL-TOPO) using the TOPO XL PCR cloning kit (Invitrogen, applying the TA rule based on the Taq polymerase's capacity of adding an extra A at the 3′ end of each DNA chain of a PCR product) as per the manufacturer's indications. Heat shock transformation into One Shot Top 10 chemically competent cells (Invitrogen) was carried out and the transformed cells were incubated in SOC medium at 37 C for 2 hrs. After that, the E. coli cells were cultured in 50 µg/ml containing LB plates overnight and the positive clones were subsequently cultured in LB broth at 37 C overnight. The plasmid alkaline extraction was done using the QIAprep spin Miniprep kit (Qiagen) as the manufacturer indicated. As a next step, verification of the probe orientation was carried out by nucleotide sequencing. Finally, transcription of the cloned DNA sequences was done to generate the RNA probe (in both sense and reverse orientations). The RNA probe was then labeled with ^32^P by using the AmpliScribe T7 High Yield Transcription Kit (EPICENTRE Biotechnologies) following the company's instructions.

### Northern blotting

To investigate the possibility that NDiV generates set of 3′-coterminal sub-genomic mRNA's during its replication, *Aedes albopictus* C6/36 cells were infected with NDiV. Three to four days after infection intracellular poly (A)-containing RNA from mock-infected and NDiV-infected cells was prepared using Dynabeads oligo(dT)_25_ (Dynal Biotech) as per the manufacturer's instructions. RNA was separated on a glyoxal-based agarose gel system and blotted on a positively charged nylon membrane (BrightStar-Plus membrane). The mRNA bands were then hybridized with an α-^32^P-multiprime-labeled RNA probe specific for NDiV at 65°C overnight (see above RNA probe generation). The membrane was then washed with low and high stringency wash solutions and the RNAs were analyzed by autoradiography. All reagents for mRNA separation, transfer and hybridization (with the exception of the RNA probe) were provided with the NorthernMax-Gly Kit (Ambion). The manufacturer's instructions were followed. A 0.5–10 Kb RNA Ladder (Invitrogen) was used as a marker set to calculate apparent molecular mass of the analyzed bands.

### Electron microscopy of virions

For electron microscopy, virus was concentrated from ICF by centrifugation at 12,000 g for 30 min at 4 C, after which 6.6% polyethylene glycol 6000 and 2.2% NaCl were added to the supernatant. After stirring for 1 h at 4 C and centrifugation at 12,000 g for 1 h, the supernatant was discarded. The virus-containing pellet was dissolved in saline-Tris-EDTA buffer, sedimented at 250,000 g for 1 h and resuspended a second time. The concentrated virus was negatively stained with 1% sodium phosphotungstic acid, pH 6.0, and examined at 100 KV using a transmission electron microscope (JEM-100CX, JEOL, Japan) [79].

### Sequencing of virion peptides

Virions were purified in a 15–50% sucrose density gradient using an SW32Ti rotor (Beckman Coulter, Inc., Fullerton,CA) at 20,000 rpm for 12–16 h at 4°C. Gradient fractions were analyzed by 16% SDS-polyacrylamide gel electrophoresis and Coomassie Brilliant Blue G staining (Fig. 2B). Protein bands were excised and either directly sequenced by automated Edman degradation (Applied Biosystems model 491cLC) or digested with lysylendopeptidase prior to HPLC purification and sequencing.

### Bioinformatics databases

Genome sizes of ssRNA+ viruses were retrieved from the NCBI Viral Genome Resource [80]. GenBank, version 178.0 [81], Pfam database, version 24.0 [34], SCOP70, version 1.75 [82], and an in-house nidovirus domain profile database [15], [54] updated in this study were used to identify putative functional domains encoded by the NDiV genome. Representatives of the nidovirus species defined according to (http://www.ictvonline.org/virusTaxonomy.asp?version=2009) plus NDiV, whose taxonomical status remains provisional, were used as detailed in Table S3. Species names of coronaviruses were taken from ICTV proposal 2008.085-122V.U that was approved by ICTV in 2009. Fields after the “_” sign in virus abbreviations represents sampling year or period.

### Basic bioinformatics analyses

The NDiV ORFs were compared with sequence databases using psi-BLAST [27], HMMer 2.3.2 [83], TMpred [84], or HHsearch [85]. Protein secondary structure predicted by Psipred [86] was included in the HHsearch-mediated profile searches. RNA secondary structure analysis was conducted using Mfold [29] and pknotsRG [30]. MUSCLE [87] was used to produce alignments of nidovirus proteins that were manually refined in poorly conserved regions. Alignment derivatives, with the least conserved columns removed [88], were prepared using BAGG [89] and were used for profile searches and phylogenetic analyses. Alignments were prepared for publication using JalView [90]. To compile and plot most graphs and conduct statistical analyses we used the R package [91].

### Identification of TRS candidates

Using the *de novo* repeat detection program RepeatScout [92] a library of perfect repeats with unit sizes ranging from four to the maximum observed size of 16 was compiled for the NDiV genome sequence. The library was filtered to retain repeats of different types according to the following constraints applied to each type separately: (i) one repeat copy must be located upstream of ORF1a, and (ii) another one must reside within the 300 nt region immediately upstream of either ORF2a, ORF3, or ORF4. Each set of the retrieved repeats was subsequently analyzed for conservation by alignment that included flanking regions of 20 nt at each side. The longest repeats with highest similarity were considered TRS candidates.

### Profile-based similarity searches

To map major nidovirus replicative proteins to pp1ab of NDiV we applied alignment-based methods. Multiple sequence alignments represent a general tool to infer both common ancestry (orthology) of residues for several related sequences (these residues form a fully occupied alignment column) and identify insertion/deletion events (corresponding to alignment columns containing gaps in selected sequences). Multiple alignments can be converted into profiles, which are statistical models that capture the degree of conservation and the likelihood to observe a certain residue or gap in each alignment column. One type of profiles are profile Hidden Markov Models (HMMs) [93] that are particularly suitable for searching for remotely related sequences (like NDiV which presumably represents a new virus family) in a probabilistic framework. They are implemented, for example, in the programs HMMer and HHsearch which were utilized in this study. A profile HMM can be compared to other HMMs or used to search for motifs in a single sequence. Due to the high degree of divergence of nidovirus sequences, we used alignments of amino acid sequences and profiles derived from these alignments to probe relation between proteins in this study.

### Phylogenetic analyses

Phylogenetic analyses were performed as described previously [94]. Bayesian posterior probability trees were compiled utilizing BEAST [95] under the WAG amino acid substitution matrix [96] using Tracer [97] to verify convergence. For the nidovirus-wide analysis, whose sampling is detailed Table S3, we used a concatenated alignment of 3CLpro, RdRp, and HEL1 including 910 aa positions and its derivative of 604 aa positions, from which least conserved columns were removed. In this analysis, the uncorrelated relaxed molecular clock approach (lognormal distribution) [98] was used as it was favored [99] over the strict molecular clock (log_10_ Bayes factor of 13.6) and equal to the relaxed molecular clock approach with exponential distribution (log_10_ Bayes Factor of 0.0). Selected internal nodes were fixed using results of separate analyses of subsets of nidoviruses. For phylogenetic analysis of the subfamily *Coronavirinae* and the family *Arteriviridae*, we used respective datasets incorporating between one and three sequences per species and including concatenated alignments of ORF1ab domains that are conserved in each of these groups. The datasets included 35 and 10 sequences for corona- and arteriviruses and consisted of 2302- and 2882-aa alignment positions, respectively. The topologies of these trees closely follow those published [51]. They were used to fix internal nodes in corona- and arterivirus clusters in the subsequent nidovirus-wide phylogenetic analysis. The exception was the basal nodes corresponding to the grouping of the *Alpha*-, *Beta*-, and *Gammacoronavirus* genera and the root of arteriviruses (EAV or SHFV), which were left unfixed. Maximum Likelihood trees were compiled utilizing the PhyML software [100]. The WAG amino acid substitution matrix and rate heterogeneity among sites (8 categories) were applied and support values for internal nodes were obtained using the non-parametric bootstrap method with 1000 replicates. Trees were rooted using domain-specific outgroups: for RdRp, three picornavirus representatives (accession numbers: NC_001489, NC_001897, NC_002058); for HEL1, four rubi-/ tetra-/ hepevirus representatives (NC_001545, NC_001990, NC_005898, NC_001434).

### Association of ExoN and large genome sizes

We sought to statistically define a genome size threshold that separates ExoN-containing from ExoN-lacking ssRNA+ viruses. To this end, we developed a custom test employing the binomial probability function and including all 43 virus groups displayed in Fig. 1. These groups consist of thousands of viruses that are believed to have emerged from a common ancestor, implying that they are not independent. Their dependence varies in virus pairs but, generally, for each virus pair is inversely proportional to the pair-wise evolutionary distance. To account for the dependence of these sequences in our test is technically challenging. To circumvent this problem, we have created a derivative of the virus dataset in which each virus family/group is represented by a single virus, in total 43 viruses. We considered the sequences of these representatives to be essentially independent due to the (extremely) large divergence that is observed, even in the most conserved genes (e.g. see Fig. 6), the lack of recognizable similarity in other genes, and the accompanied gene loss and gain.

For a given genome size threshold, ssRNA+ viruses were partitioned into two groups (below and above that threshold) and the value of the binomial density function was calculated for both groups using information on the presence or absence of ExoN. The final probability of the test is the product of the binomial probabilities for the two groups. We used a binomial success probability of 4/43 since four out of the 43 ssRNA+ virus lineages (NDiV, toro-/bafiniviruses, coronaviruses, and roniviruses) employ ExoN. The test was applied to each possible threshold separating two unique ssRNA+ genome sizes, in total – 42 thresholds. The threshold of ∼20 kb, between the genome sizes of NDiV and closteroviruses, gave the lowest probability to observe the ExoN association by chance. We consider the obtained value (10^−6^) as an underestimate of the true probability that should be calculated by taking into account the sequence dependence *and* all viruses in the 43 groups, which without exception conform to the ExoN distribution observed in the selected virus representatives used now.

### Accession numbers

RefSeq accession numbers of proteins referred to in the text for a selection of prototype nidoviruses are: 3C-like proteinase (EAV: NP_705584, SARS-CoV: NP_828863, WBV: YP_803213, GAV: YP_001661453), RNA-dependent RNA polymerase (EAV: NP_705590, SARS-CoV: NP_828869, WBV: YP_803213, GAV: YP_001661452), superfamily 1 helicase (EAV: NP_705591, SARS-CoV: NP_828870, WBV: YP_803213, GAV: YP_001661452), exoribonuclease (SARS-CoV: NP_828871, WBV: YP_803213), N7-methyltransferase (SARS-CoV: NP_828871), uridylate-specific endonuclease (EAV: NP_705592, SARS-CoV: NP_828872, WBV: YP_803213) and 2′-O-methyltransferase (SARS-CoV: NP_828873, WBV: YP_803213).

## Supporting Information

Figure S1 **Cloning and sequencing details.** (A) To obtain RT-PCR products containing unknown NDiV sequences upstream of the previously sequenced region of the genome the following was done: cDNAs of the NDiV RNA were converted into ds cDNAs, which were digested by restriction enzymes and subsequently ligated to an anchor DNA using those existing restriction sites. For a detailed explanation of each procedure please read the “Genome cloning and sequencing” section of Materials and Methods. (B) Semi-nested PCR was conducted for the anchored ds cDNA of NDiV using one pUC19 specific sense primer (primer pUC119-scaI208 was used for the *Sca*I-digested sample and primer pUC19-EcoRI227 was used for the samples digested with all the other restriction enzymes) and two reverse gene-specific primers (GSPs) of NDiV for each experiment. The PCR products contained the unknown sequence between GSP and anchor. This process was repeated eight times, and this protocol allowed us to read a total of 7164 bp. The name of each restriction enzyme is followed by its position written in brackets as explained below. NDiV-RACE117-99R1>NDiV-RACE74-54R2 means “primer for first PCR>primer for nested PCR”. 878 bp>836 bp means “size of the first PCR>size for the nested PCR”. If 1, 2, or 3 asterisks are in brackets, non-specific cuts took place with the following details: (*) non-specific cut and ligation occurred at 4433 bp; (**) non-specific anchoring at 513 bp; (***) In the eighth step, anchor DNA of *Bam*HI and *Hind*III attached to same location, and it was suggested that reverse transcription stopped there. The GeneRacer (TM) Kit (Invitrogen) was used to read the remaining 205 bp toward the 5′-end of genomic RNA.
(PDF)

Figure S2 **Hydrophobicity plots and secondary structure predictions for (presumed) NDiV structural proteins.** Hydrophobicity was calculated using TMpred for the pp1a replicase precursor (ORF1a; A) that served as a control for four (putative) virion proteins p2a (ORF2a; B), p2b (ORF2b; C), p3 (ORF3; D) and p4 (ORF4; E). Horizontal dashed lines depict the threshold (value of 500) for significant association with transmembrane helices. On top of the plots for the structural proteins, Jpred-mediated secondary structure predictions are shown. Predicted alpha helices and beta strands are highlighted in red and green, respectively.
(PDF)

Figure S3 **Association of ExoN with large nidovirus genomes and progress in nidovirus genomics.** At the X axis, a timeline of nidovirus genome sequencing is plotted. It starts in 1991 when the first pair of genome sequences for both small and large nidoviruses, arterivirus (EAV) and coronavirus (IBV), respectively, became available. The genome size difference (gap) between these viruses was ∼14.9 kb. All subsequent time points were selected because new nidoviruses with either larger (for small nidoviruses) or smaller (for large nidoviruses) genomes were released in these years. (For the purpose of this analysis, NDiV was treated as a large nidovirus). As a result, the genome size gap shrunk, in total six times since 1991 (three times in 1993). Currently, the gap that remains is ∼4.5 kb (the arterivirus SHFV vs. NDiV). Large nidoviruses are assumed to have acquired a unique genomic region during the expansion of their genome. It includes ExoN and OMT, and some other genes, like the NMT that is found in some large nidoviruses. This region may also include additional domains, due to the thus far incomplete characterization of the nidovirus proteome; they might include one or more with the phyletic distribution characteristic of ExoN and OMT. Understandably, as the genome size gap between large and small nidoviruses has been shrinking due to the discovery of new nidoviruses, the probability that such genes exist is decreasing. Likewise, the share of the size of the ExoN and OMT domains in the total genome size gap could be considered a measure of confidence for the role of these genes in nidovirus genome expansion. At the Y axis, the growth of this share is plotted; it gradually increased from ∼12% in 1991 (EAV vs. IBV) to ∼35% in 2011 (SHFV vs. NDiV). By far the biggest increase (∼17%), and hence the largest gain in support for the role of ExoN in nidovirus genome expansion, was achieved by the sequence analysis of the NDiV genome. The above numbers outline a trend and this analysis should not be confused with a probabilistic framework.
(PDF)

Table S1 **Affinity of corona- and toro-/bafiniviruses.**
(RTF)

Table S2 **Primers used to sequence the 5′-end of the NDiV genome.**
(DOC)

Table S3 **Genome sequences of a representative set of the Nidovirus species.**
(DOC)

Text S1 **Sequence similarity-based clustering of corona- and toroviruses.**
(RTF)
