# Supplementary figures and images for: Discovery of the First Insect Nidovirus, a Missing Evolutionary Link in the Emergence of the Largest RNA Virus Genomes
Source: PLoS Pathog. 2011 Sep 8;7(9):e1002215. doi: 10.1371/journal.ppat.1002215 (PMC3169540; doi:10.1371/journal.ppat.1002215)

A

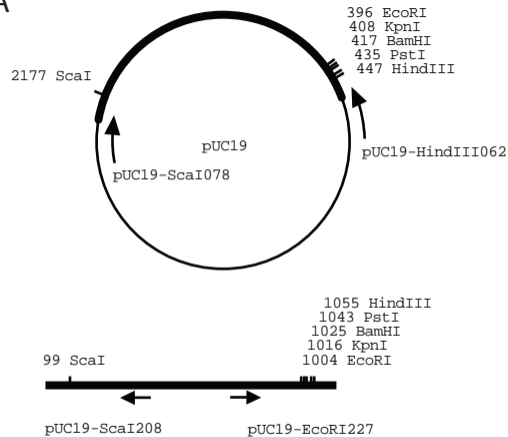

B

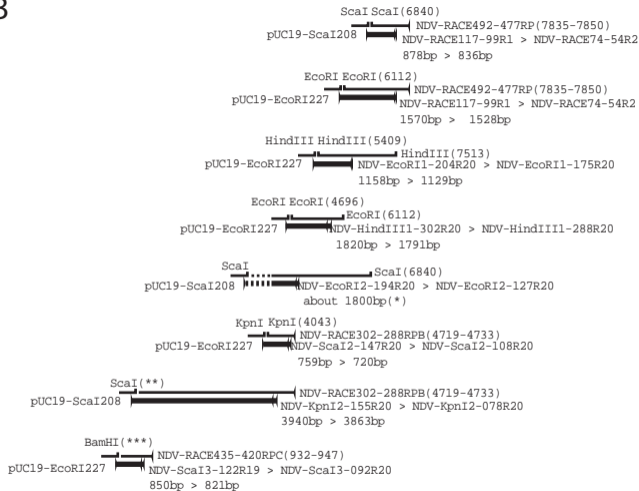

Supplement: Figure S1 — Cloning and sequencing details. (A) To obtain RT-PCR products containing unknown NDiV sequences upstream of the previously sequenced region of the genome the following was done: cDNAs of the NDiV RNA were converted into ds cDNAs, which were digested by restriction enzymes and subsequently ligated to an anchor DNA using those existing restriction sites. For a detailed explanation of each procedure please read the “Genome cloning and sequencing” section of Materials and Methods. (B) Semi-nested PCR was conducted for the anchored ds cDNA of NDiV using one pUC19 specific sense primer (primer pUC119-scaI208 was used for the ScaI-digested sample and primer pUC19-EcoRI227 was used for the samples digested with all the other restriction enzymes) and two reverse gene-specific primers (GSPs) of NDiV for each experiment. The PCR products contained the unknown sequence between GSP and anchor. This process was repeated eight times, and this protocol allowed us to read a total of 7164 bp. The name of each restriction enzyme is followed by its position written in brackets as explained below. NDiV-RACE117-99R1>NDiV-RACE74-54R2 means “primer for first PCR>primer for nested PCR”. 878 bp>836 bp means “size of the first PCR>size for the nested PCR”. If 1, 2, or 3 asterisks are in brackets, non-specific cuts took place with the following details: (*) non-specific cut and ligation occurred at 4433 bp; (**) non-specific anchoring at 513 bp; (***) In the eighth step, anchor DNA of BamHI and HindIII attached to same location, and it was suggested that reverse transcription stopped there. The GeneRacer (TM) Kit (Invitrogen) was used to read the remaining 205 bp toward the 5′-end of genomic RNA. (PDF) [file ppat.1002215.s001.pdf]

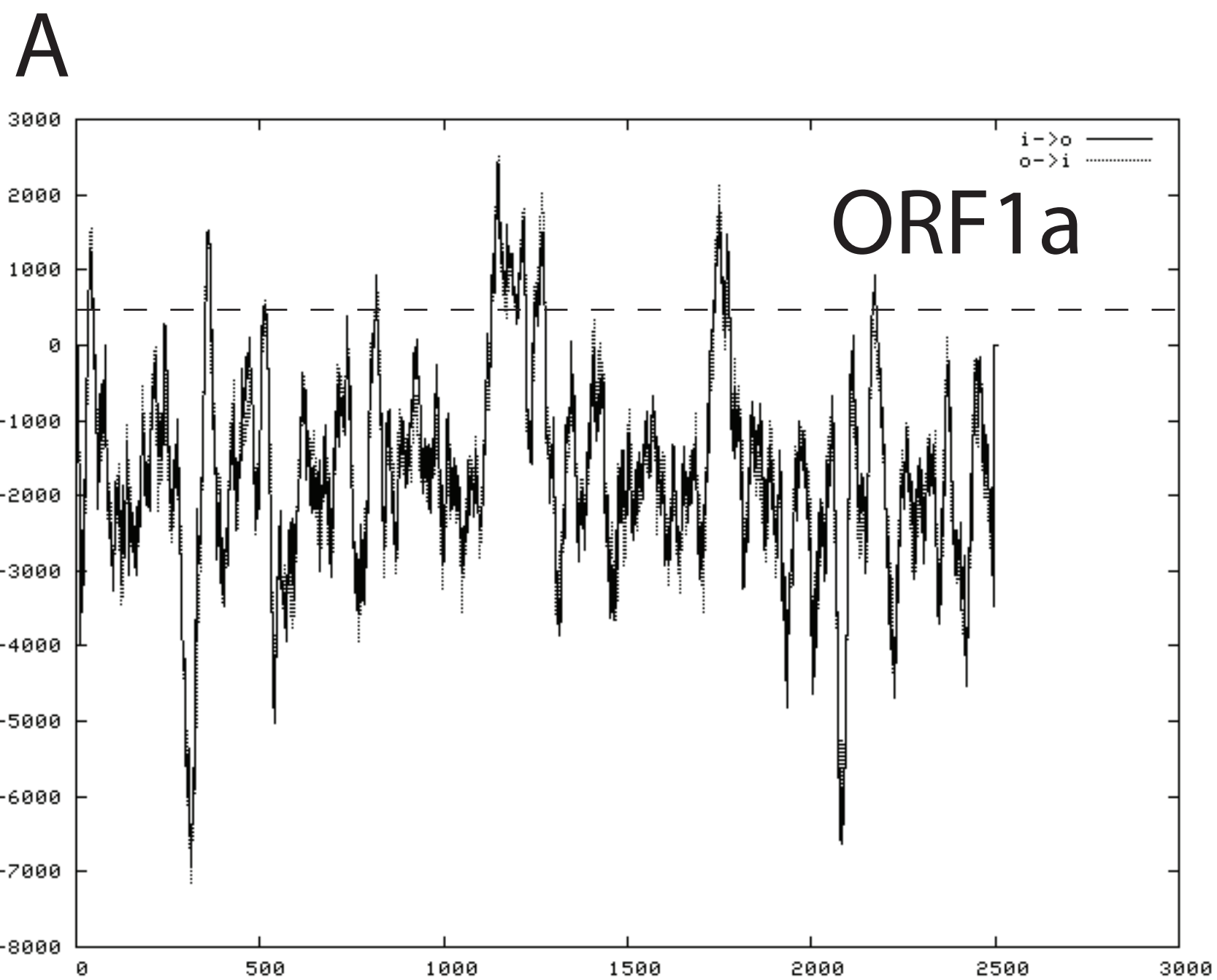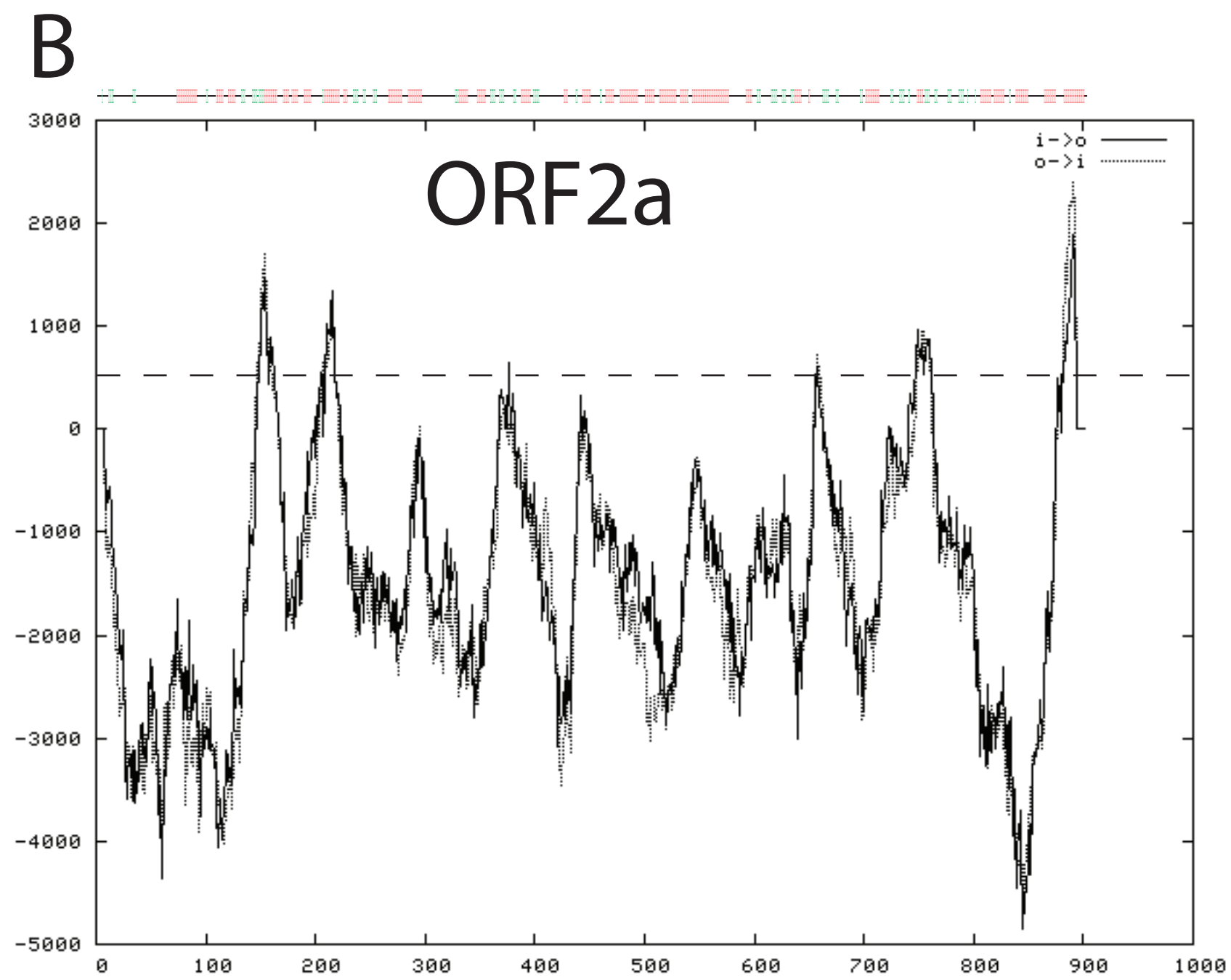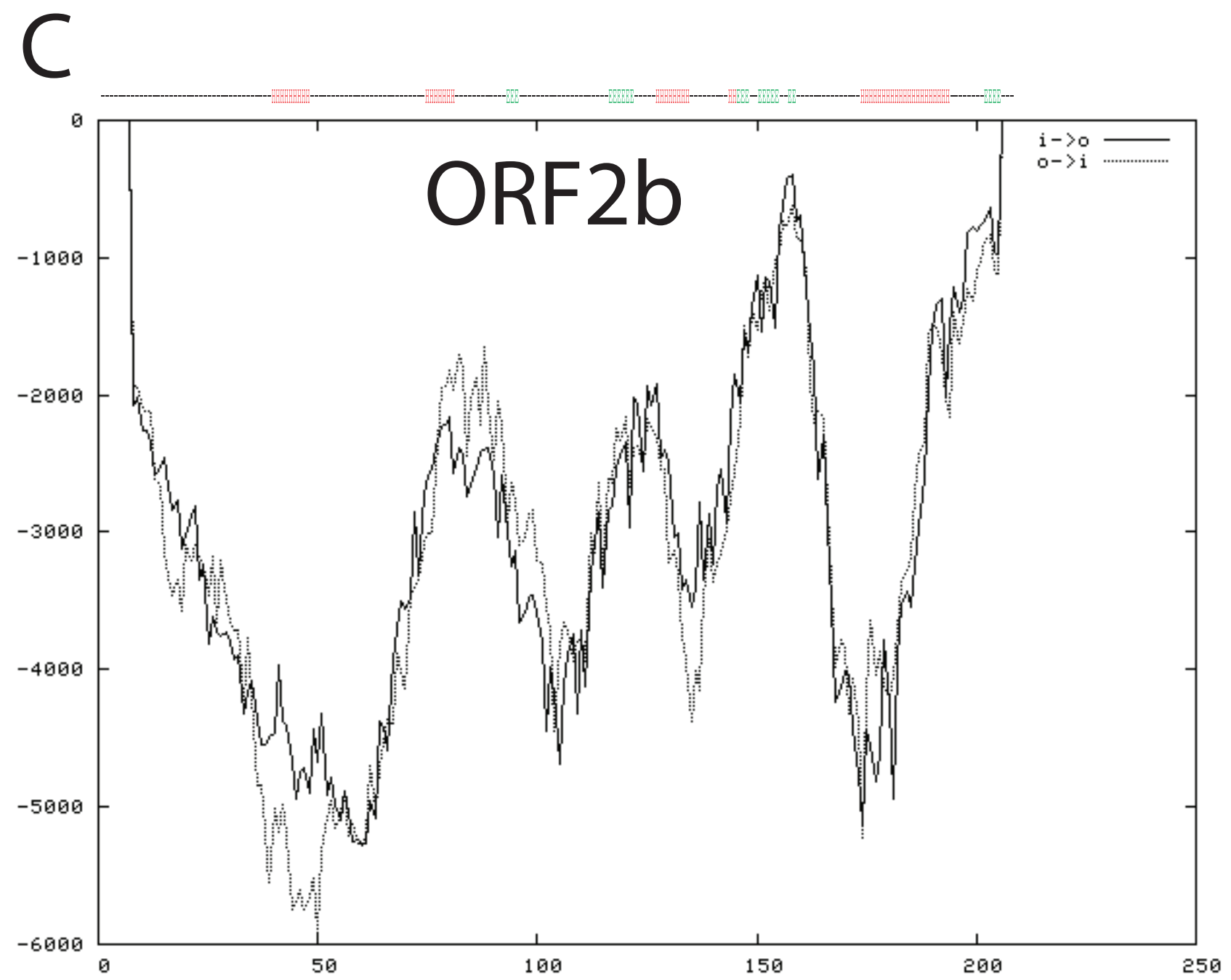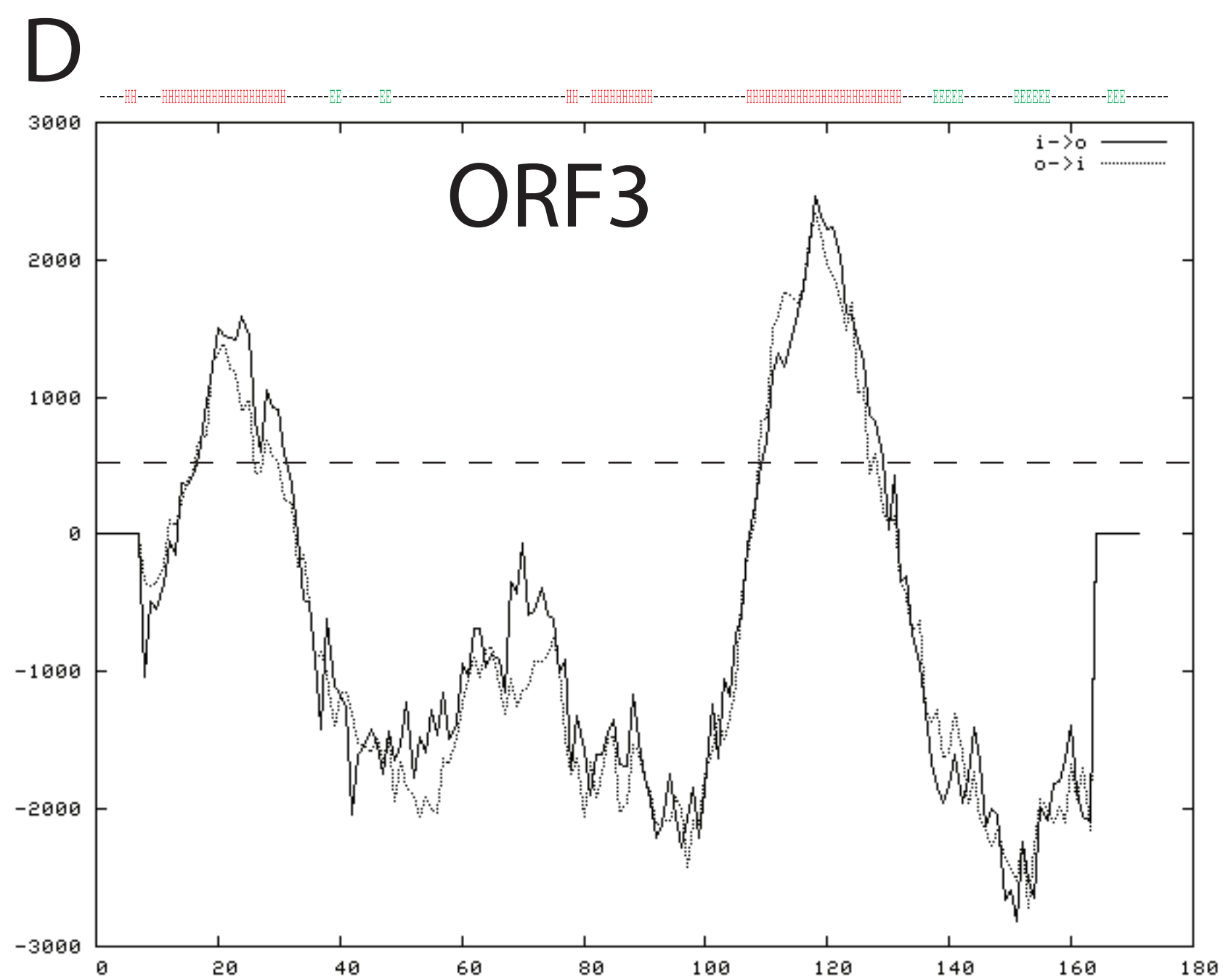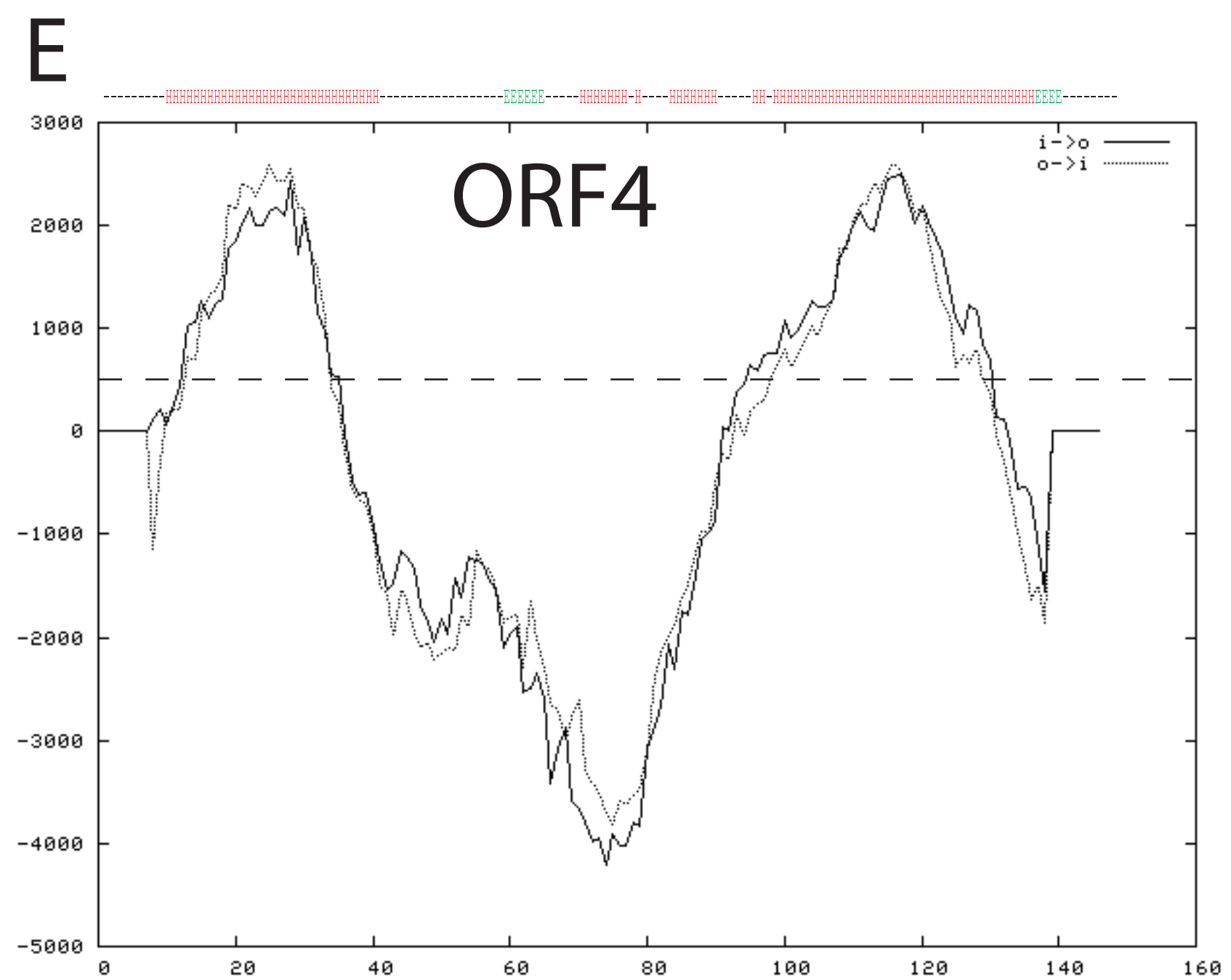

Supplement: Figure S2 — Hydrophobicity plots and secondary structure predictions for (presumed) NDiV structural proteins. Hydrophobicity was calculated using TMpred for the pp1a replicase precursor (ORF1a; A) that served as a control for four (putative) virion proteins p2a (ORF2a; B), p2b (ORF2b; C), p3 (ORF3; D) and p4 (ORF4; E). Horizontal dashed lines depict the threshold (value of 500) for significant association with transmembrane helices. On top of the plots for the structural proteins, Jpred-mediated secondary structure predictions are shown. Predicted alpha helices and beta strands are highlighted in red and green, respectively. (PDF) [file ppat.1002215.s002.pdf]

OMT+Exon share [%]

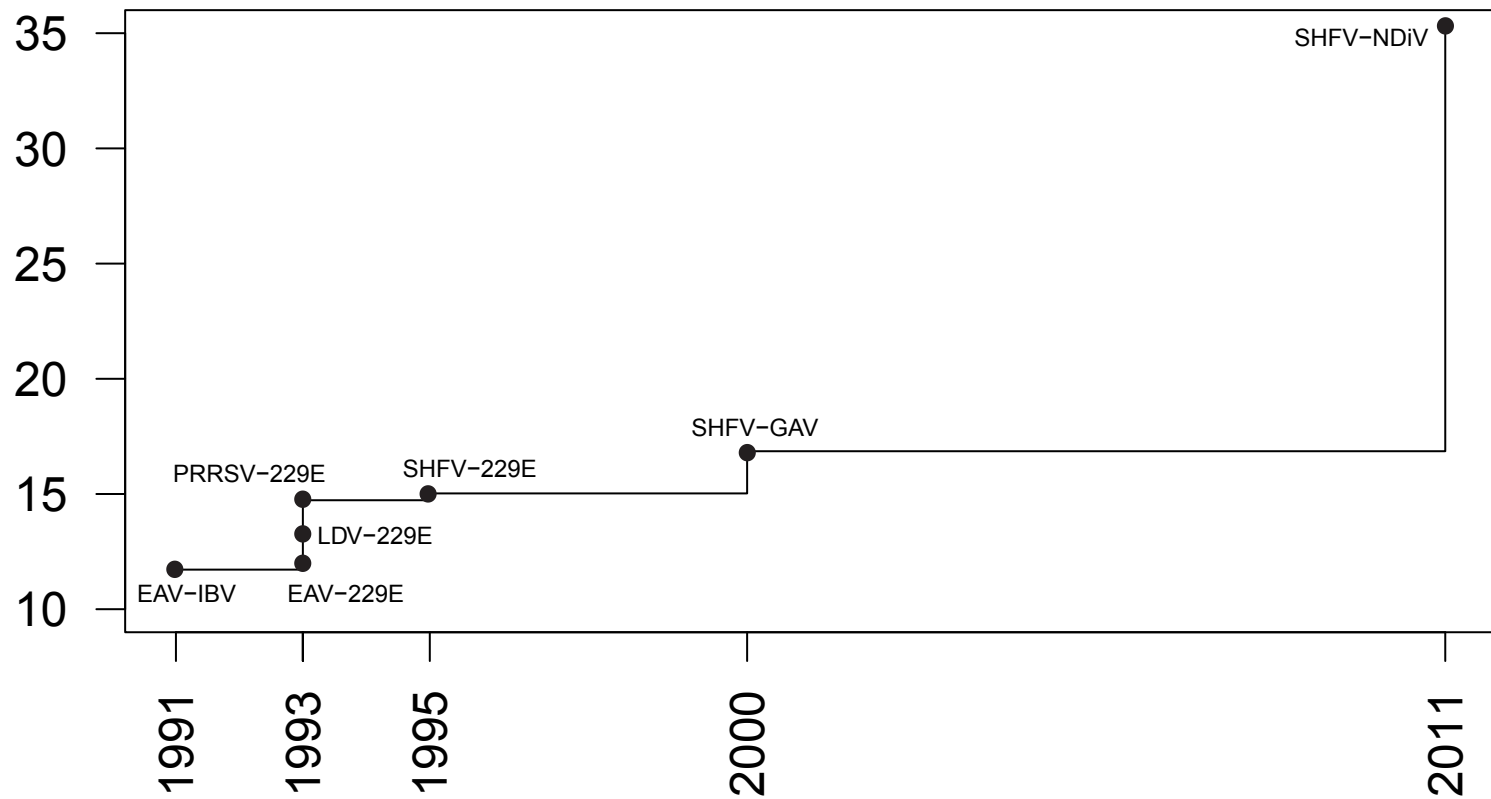

Supplement: Figure S3 — Association of ExoN with large nidovirus genomes and progress in nidovirus genomics. At the X axis, a timeline of nidovirus genome sequencing is plotted. It starts in 1991 when the first pair of genome sequences for both small and large nidoviruses, arterivirus (EAV) and coronavirus (IBV), respectively, became available. The genome size difference (gap) between these viruses was ∼14.9 kb. All subsequent time points were selected because new nidoviruses with either larger (for small nidoviruses) or smaller (for large nidoviruses) genomes were released in these years. (For the purpose of this analysis, NDiV was treated as a large nidovirus). As a result, the genome size gap shrunk, in total six times since 1991 (three times in 1993). Currently, the gap that remains is ∼4.5 kb (the arterivirus SHFV vs. NDiV). Large nidoviruses are assumed to have acquired a unique genomic region during the expansion of their genome. It includes ExoN and OMT, and some other genes, like the NMT that is found in some large nidoviruses. This region may also include additional domains, due to the thus far incomplete characterization of the nidovirus proteome; they might include one or more with the phyletic distribution characteristic of ExoN and OMT. Understandably, as the genome size gap between large and small nidoviruses has been shrinking due to the discovery of new nidoviruses, the probability that such genes exist is decreasing. Likewise, the share of the size of the ExoN and OMT domains in the total genome size gap could be considered a measure of confidence for the role of these genes in nidovirus genome expansion. At the Y axis, the growth of this share is plotted; it gradually increased from ∼12% in 1991 (EAV vs. IBV) to ∼35% in 2011 (SHFV vs. NDiV). By far the biggest increase (∼17%), and hence the largest gain in support for the role of ExoN in nidovirus genome expansion, was achieved by the sequence analysis of the NDiV genome. The above numbers outline a trend and this analys [file ppat.1002215.s003.pdf]
