# Supplementary material for: Discovery of the First Insect Nidovirus, a Missing Evolutionary Link in the Emergence of the Largest RNA Virus Genomes
Source: PLoS Pathog. 2011 Sep 8;7(9):e1002215. doi: 10.1371/journal.ppat.1002215 (PMC3169540; doi:10.1371/journal.ppat.1002215)
Supplement: Table S1 — Affinity of corona- and toro-/bafiniviruses. (RTF) [file ppat.1002215.s004.rtf]

Table S1. Affinity of corona- and toro-/bafinivirusesa. 

coronavirus
protein profileb	targetc	
	coronad	toro/bafini	roni	arteri	
					
ADRP	e-105	e-25	330	34	
PL2pro	e-126	40	110	160	
3CLpro	e-244	24	88	43	
primase	e-152	4	27	200	
RdRp	~0	e-14	0.004	0.079	
HEL1	e-227	e-6	e-13	0.81	
ExoN	e-244	0.008	0.62	38	
NMT	e-204	22	13	46	
NendoU	e-133	e-4	23	e-4	
OMT	e-245	e-8	0.29	57	
Se	~0	0.005	100	240	
M	e-158	1	990	12	
E	e-37	56f	250	11	
N	e-247	12000	7000	2800	

a HMMer profile searches (global profile against local sequence) were used to determine closest nidovirus relatives of a selection of proteins expressed by coronaviruses. E-values are based on a database size of 12000 according to the size of the Pfam (version 24.0, October 2009). The best hit against the profile of the coronavirus protein alignment is indicated in italic and unique, significant (E-value <= 1) best hits against toro-/bafini- or roniviruses in bold
b a profile of an alignment containing 17 coronavirus species was used. ADRP, ADP-ribose-1''-phosphatase; PL2pro, papain-like proteinase 2; 3CLpro, 3C-like proteinase; RdRp, RNA-dependent RNA polymerase; HEL1, superfamily 1 helicase; ExoN, 3'-to-5'exoribonuclease; NMT, N7-methyltransferase; NendoU, uridylate-specific endonuclease;  OMT, 2'-O-methyltransferase. 
c numbers represent E-values of a HMMer search against the coronavirus protein profile  
d E-values of hits against coronaviruses itself are shown for comparison
e only the C-terminal part of the coronavirus S protein alignment (S2) was used as a profile
f hit is against the torovirus M protein
