# Supplementary material for: Discovery of the First Insect Nidovirus, a Missing Evolutionary Link in the Emergence of the Largest RNA Virus Genomes
Source: PLoS Pathog. 2011 Sep 8;7(9):e1002215. doi: 10.1371/journal.ppat.1002215 (PMC3169540; doi:10.1371/journal.ppat.1002215)
Supplement: Table S2 — Primers used to sequence the 5′-end of the NDiV genome. (DOC) [file ppat.1002215.s005.doc]

**Table S2.** Primers used to sequence the 5’-end of the NDiV genome.

| **Primer** | **Sequence** | **Application** |
| --- | --- | --- |
| NDiV-RACE492-477RP | AAATCCAAAGGGTGCT | cDNA generation |
| NDiV-RACE117-99R1 | GCGTTCAAAATAGCCAAGT | Semi-nested PCR |
| NDiV-RACE74-54R2 | TAGTAATAGCCTTCAGGATCG | Semi-nested PCR |
| NDiV-EcoRI1-204R20 | ATCGAGTGTGTCTGGAGTAG | Semi-nested PCR |
| NDiV-EcoRI1-175R20 | TCAAATAGCGTACGAGTTCA | Semi-nested PCR |
| NDiV-HindIII1-288R20 | AAGATGATGGAGCTAAGGAT | Semi-nested PCR |
| NDiV-HindIII1-302R20 | TGTGGGGGATTGTAAAGATG | Semi-nested PCR |
| NDiV-EcoRI2-127R20 | GGATGTAAGCTGATATGTGG | Semi-nested PCR |
| NDiV-EcoRI2-194R20 | TTTGTTTAGTTCCGTGTCGT | Semi-nested PCR |
| NDiV-ScaI2-108R20 | TGTGAAATTGAGGGGTTTGA | Semi-nested PCR |
| NDiV-ScaI2-147R20 | ATTAGAGGGTTAATGGCAAC | Semi-nested PCR |
| NDiV-RACE302-288RPB | AGAAGCCCCTTACCA | cDNA generation |
| NDiV-KpnI2-078R20 | CTGGTGCAGACGTACGGAAT | Semi-nested PCR |
| NDiV-KpnI2-155R20 | TTAGGTAGTTTGGTCGTTGT | Semi-nested PCR |
| NDiV-RACE435-420RPC | TCGCTTACTGCTTTCT | cDNA generation |
| NDiV-ScaI3-122R19 | GAAAAATGTTTAGGCGAGA | Semi-nested PCR |
| NDiV-ScaI3-092R20 | GCAAAAACTGGTGTTTGATA | Semi-nested PCR |
| pUC19-EcoRI227 | ACAGATGCGTAAGGAGAAAA | Semi-nested PCR |
| pUC19-ScaI208 | AACGCTGGTGAAAGTAAAAG | Semi-nested PCR |
| pUC19-HindIII062 | TATGCTTCCGGCTCGTATGT | Anchor |
| pUC19-ScaI078 | AGTAAGTTGGCCGCAGTGT | Anchor |
| NDiVpolyA-R | TTACCTGTAATGCCAAGCGC | Northern blot* |
| NDiV19733-F | CGCCTGTAAGAGAGATTGTA | Northern blot* |
