# Supplementary material for: Discovery of the First Insect Nidovirus, a Missing Evolutionary Link in the Emergence of the Largest RNA Virus Genomes
Source: PLoS Pathog. 2011 Sep 8;7(9):e1002215. doi: 10.1371/journal.ppat.1002215 (PMC3169540; doi:10.1371/journal.ppat.1002215)
Supplement: Table S3 — Genome sequences of a representative set of the Nidovirus species. (DOC) [file ppat.1002215.s006.doc]

**Table S3.** Genome sequences of a representative set of the Nidovirus species.

| species name^a^ |  | virus abbreviation^b^ | (sub)family | acc. number |
| --- | --- | --- | --- | --- |
| Nam Dinh virus |  | NDiV_01-03 | - | - |
| Gill-associated virus |  | GAV_96 | Ronivirus | AF227196 |
| Yellow head virus |  | YHV_98 | Ronivirus | EU487200 |
| White bream virus |  | WBV-DF24_00 | Torovirus | NC_008516 |
| Equine torovirus |  | EToV-Berne_72 | Torovirus | X52374 |
| Bovine torovirus |  | BToV-Breda1_79 | Torovirus | NC_007447 |
| Human coronavirus 229E |  | HCoV-229E_65 | Coronavirus | NC_002645 |
| Human coronavirus NL63 |  | HCoV-NL63_02 | Coronavirus | DQ445911 |
| *Miniopterus* bat coronavirus 1 |  | Mi-BatCoV-1A_05 | Coronavirus | NC_010437 |
| *Rhinolophus* bat coronavirus HKU2 |  | Rh-BatCoV-HKU2_06 | Coronavirus | NC_009988 |
| *Miniopterus* bat coronavirus HKU8 |  | Mi-BatCoV-HKU8_05 | Coronavirus | NC_010438 |
| *Scotophilus* bat coronavirus 512 |  | Sc-BatCoV-512_05 | Coronavirus | DQ648858 |
| Porcine epidemic diarrhoea virus |  | PEDV-CV777_77 | Coronavirus | NC_003436 |
| Geselavirus |  | FCoV_79 | Coronavirus | NC_007025 |
| SARS-related coronavirus |  | SARS-HCoV_03 | Coronavirus | AY345988 |
| *Tylonycteris* bat coronavirus HKU4 |  | Ty-BatCoV-HKU4_04 | Coronavirus | EF065505 |
| *Pipistrellus* bat coronavirus HKU5 |  | Pi-BatCoV-HKU5_04 | Coronavirus | EF065509 |
| *Rousettus* bat coronavirus HKU9 |  | Ro-BatCoV-HKU9_05 | Coronavirus | EF065513 |
| Human coronavirus HKU1 |  | HCoV-HKU1_04 | Coronavirus | AY884001 |
| Betacoronavirus 1 |  | HCoV-OC43_67 | Coronavirus | AY585228 |
| Murine coronavirus |  | MHV-A59_59 | Coronavirus | AY700211 |
| Avian coronavirus |  | IBV-Beaud_35 | Coronavirus | NC_001451 |
| Beluga whale coronavirus SW1 |  | BWCoV-SW1_06 | Coronavirus | EU111742 |
| Equine arteritis virus |  | EAV-CW_96 | Arterivirus | AY349167 |
| Simian hemorrhagic fever virus |  | SHFV_64 | Arterivirus | NC_003092 |
| Lactate dehydrogenase-elevating virus |  | LDV-P_71 | Arterivirus | U15146 |
| Porcine respiratory and reproductive syndrome virus, North American type |  | PRRSV-NA_95 | Arterivirus | AF176348 |
| Porcine respiratory and reproductive syndrome virus, European type |  | PRRSV-LV_91 | Arterivirus | M96262 |

^a^ species names of coronaviruses taken from ICTV proposal 2008.085-122V.U that was approved by ICTV in 2009.

^b^ field after the “_” sign represents sampling year or period
