# Supplementary material for: Discovery of the First Insect Nidovirus, a Missing Evolutionary Link in the Emergence of the Largest RNA Virus Genomes
Source: PLoS Pathog. 2011 Sep 8;7(9):e1002215. doi: 10.1371/journal.ppat.1002215 (PMC3169540; doi:10.1371/journal.ppat.1002215)
Supplement: Text S1 — Sequence similarity-based clustering of corona- and toroviruses. (RTF) [file ppat.1002215.s007.rtf]

Text S1. Sequence similarity-based clustering of corona- and toroviruses
There is a consensus in the field that coronaviruses and toro/bafiniviruses are nidoviral sister lineages. This relationship has been codified in nidovirus taxonomy with these two groups of viruses forming the two subfamilies in the family Coronaviridae. Yet, prior phylogenetic analyses using either RdRp or HEL1 were not as conclusive about this clustering [50], which prompted us to verify it using an alternative approach. We sought to use similarity of domains that are conserved in the subfamily Coronavirinae and (partly) shared with other nidoviruses to rank toro/bafiniviruses, roniviruses and arteriviruses in relation to the subfamily Coronavirinae. We compiled HMMER profiles for 14 protein domains of 17 coronaviruses, representing replicative proteins (10 domains: ADP-ribose-1''-phosphatase (ADRP), papain-like proteinase 2 (PL2pro), 3CLpro, primase, RdRp, HEL1, ExoN, NMT, NendoU and OMT) and virion proteins (4 domains: S, M, E and N) that together account for ~45% of the ~29kb genome. They were compared in the global profile vs. local sequence mode against products of all ORFs encoded by a representative set of nidoviruses. 
The obtained E-values of the top hits for four phylogenetic groups, corona-, toro/bafini-, roni, and arteriviruses were compared for each protein domain (Table S1). Eight domains (6 replicative and 2 virion domains) produced significant hits outside coronaviruses: all 8 with toro/bafiniviruses, and 4 different domains with, separately, roniviruses and arteriviruses. Based on the best hit E-values, toro/bafiniviruses were ranked the top for 6 domains (ADRP, RdRp, ExoN, OMT, S, and M), shared the top spot for one domain with arteriviruses (NendoU) and were ranked second after roniviruses for the HEL1 domain. According to another analysis that is presented in Fig. 5C, corona- and roniviruses but not toroviruses also share an NMT domain. However, this conservation was too remote to be identified by the HMMER-based analysis and it was not included in Table S1. Regardless of considerations involving the NMT domain, the presented results confirm a special sequence affinity between coronaviruses and toro/bafiniviruses among nidoviruses. 
